# General anesthetic modulation of a pentameric ligand-gated ion channel explored with simulations

**DOI:** 10.1016/j.bpj.2026.05.007

**Published:** 2026-05-15

**Authors:** Adam J. Dymke, Bogdan Lev, Toby W. Allen

**Affiliations:** 1 School of Science, RMIT University, Melbourne, VIC, Australia

**Keywords:** pentameric ligand-gated ion channel, general anesthetic, propofol, molecular dynamics, metadynamics, free energy

## Abstract

General anesthetics are known to modulate pentameric ligand-gated ion channels (pLGICs), including GABA_A_, glycine, serotonin, and glutamate receptors, among others. The bacterial (*Gloeobacter*) ion channel GLIC captures anesthetic modulation while providing high-resolution structures in each of its distinct functional conformations, allowing for molecular definition of state-dependent binding and quantification of allosteric mechanisms. This study uses molecular dynamics, metadynamics, and free energy simulations to calculate the free energy surfaces of propofol binding to open and closed channels. We observe distinct binding sites in the pore, within and between transmembrane domain subunits, as well as in the extra-cellular domain, that differ in location and free energies between open and closed states. Each transmembrane site was seen to bifurcate into two adjacent sites, with overlapping residues that agree with X-ray crystallography, but with other residues identified as strongly interacting that provide predictions for experimental verification. The investigation revealed distinct pathways between intra-subunit, inter-subunit, and pore sites in the open and closed states. Multiple propofol binding to neighboring subunits was seen to modify the binding surface and lead to a third inter-subunit site within the closed state. Calculated free energy changes are consistent with previous suggestions of bimodal modulation via competing sites that act to inhibit or potentiate the channel. Inhibition via pore binding involves pore occlusion by propofol, with minor changes to pore shape and size, notably tending to close the channel when propofol is bound. Binding to intra-subunit sites was shown to thermodynamically favor the open state, in agreement with experimental evidence for potentiation. Calculations of binding to inter-subunit sites were found to involve opposing effects from the two constituent sites, leading to no clear net shift in equilibrium. These observations offer molecular explanations for the effects of propofol that are likely to apply to other pLGICs, assisting in the development of improved anesthetics with better safety margins in future.

## Significance

Pentameric ligand-gated ion channels (pLGICs) are the main target for general anesthetics in the central nervous system. Structural resolution of these proteins has provided a platform for understanding general anesthesia, the mechanisms of which have been debated for over a century. Advanced biomolecular simulations and free energy calculations offer the ability to extract molecular-level information on where general anesthetic molecules bind, how they modify protein structure, and how they influence ion channel activity via preferential binding to one conformational state over another. The results complement structural and functional experiments by revealing previously undescribed interactions and pathways, together with quantitative measures of allosteric modulation of ion channel function. The findings may enable new studies that can target pLGICs with novel anesthetics.

## Introduction

General anesthesia is essential for almost all surgical procedures, relying on the ability to sedate and immobilize patients safely. The dominant mechanism of general anesthesia appears to involve modulation of pentameric ligand-gated ion channels (pLGICs) in the central nervous system.^1^ Specifically, Cys-loop receptors (pLGICs containing a Cys-loop structural motif) are known to be general anesthetic targets.^2^^,^^3^ In particular, γ-aminobutyric acid A receptors (GABA_A_R) have been implicated,^4^^,^^5^ with residues of individual GABA_A_R subunits identified as key determinants of anesthetic sensitivity.^6^^,^^7^ These proteins are located at synaptic junctions and play a vital role in neural signal transmission. Anesthesia involves a decrease in signal propagation in the nervous system^8^ by potentiating inhibitory Cl^−^-selective channels, such as GABA_A_R,^4^^,^^5^ while at the same time inhibiting excitatory Na^+^-selective channels (e.g., nAChR^9^). The specific interaction between anesthetics and these targets can vary depending on the type of anesthetic, highlighting the complex nature of general anesthesia and the need for a molecular definition of their mechanisms.

**Figure 1:**
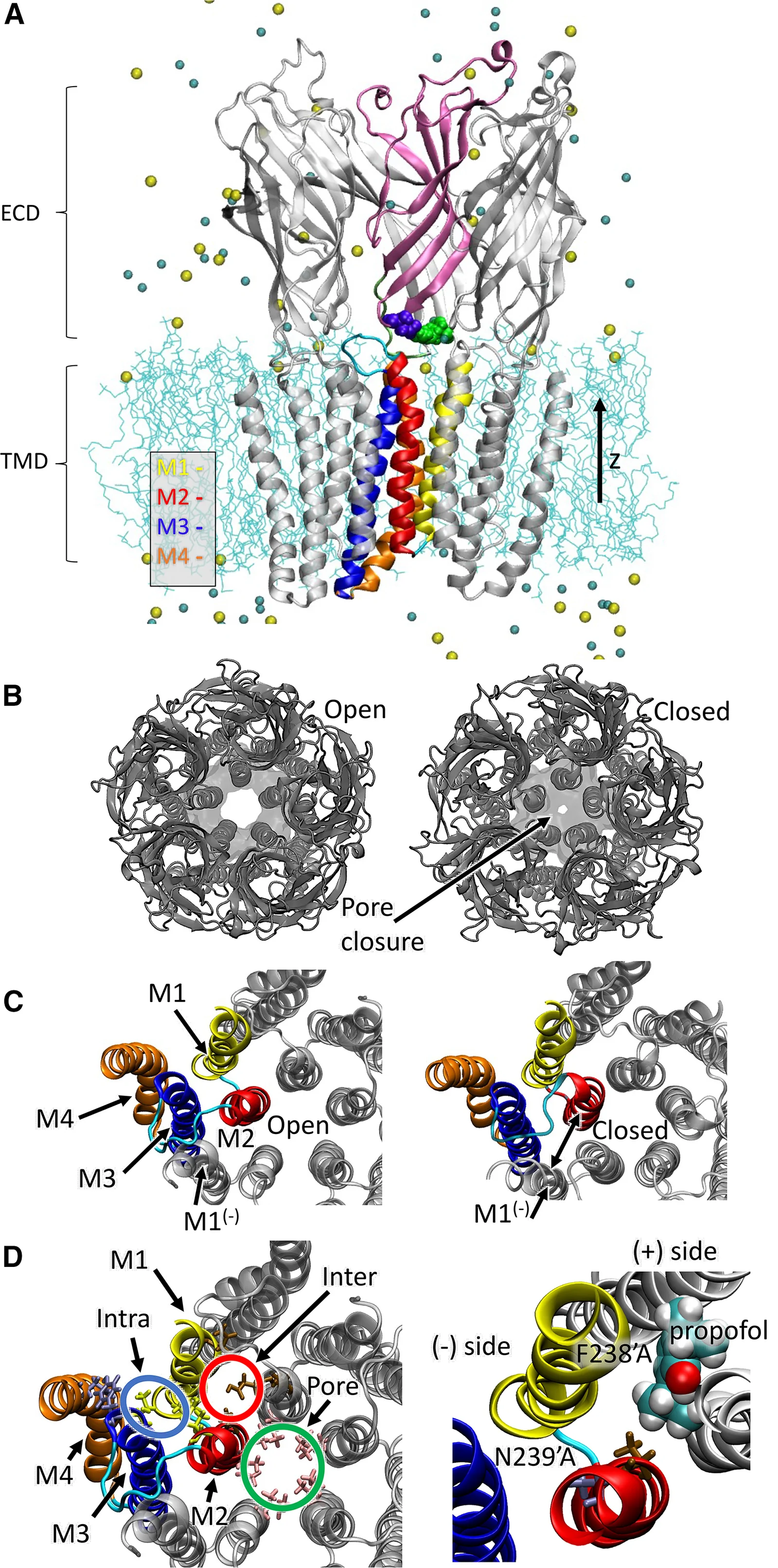
Structure of the GLIC functional states. (A) MD system showing transmembrane helices labeled M1 (yellow), M2 (red), M3 (blue), and M4 (orange). Water is not shown. Highlighted with green (R192) and blue (D32) balls is the salt bridge R192-D32 that plays a key role in gating. (B) Open- and closed-state GLIC showing pore constriction. (C) M2 movement during gating (O, left; C, right), with changing M2-M1^(−)^ distance indicated. (D). TMD sites in the O GLIC structure (left). Right: F238A/N239A mutation, with propofol included in the inter-subunit site.

X-ray crystallography (e.g., Miller and Aricescu^10^) of the human GABA_A_R channel has suggested a highly conserved structure to other pLGICs (with low Cα RMSD of <2Å over the entire protein), including bacterial channels (ELIC^11^ and GLIC^12^^,^^13^). Each member of the pLGIC superfamily is composed of five identical or homologous subunits, radially arranged around a central pore (e.g., Figure 1A for GLIC^14^). Each subunit comprises an N-terminal extra-cellular domain (ECD), consisting of a 10-strand β sheet sandwich, followed by a transmembrane domain (TMD) consisting of four transmembrane helices (M1–M4; Figure 1A). There exist neurotransmitter- or agonist-binding sites at the ECD subunit interfaces, allosterically activating the channel’s TMD opening, as has been studied with both experiment^15^^,^^16^^,^^17^^,^^18^^,^^19^^,^^20^^,^^21^ and computation.^22^^,^^23^^,^^24^^,^^25^^,^^26^

To explore modulation by anesthetics, pLGIC structures in both the activated/open (O) and closed (C) functional forms are required. High-resolution structures of several pLGICs have been solved with X-ray crystallography or cryogenic electron microscopy (cryo-EM) in recent years, including for the β_3_ GABA_A_ receptor (GABA_A_R),^10^ the α_1_-glycine receptor (GlyR),^27^^,^^28^ the 5-hydroxytryptamine receptor (5-HT_3_),^29^ the α_4_β_2_ nicotinic acetylcholine receptor (nAChR),^30^ the glutamate-gated chloride channel (GluCl),^31^^,^^32^ the bacterial *Erwinia chrysanthemi* channel ELIC,^11^ and the bacterial (*Gloeobacter*) ion channel (GLIC).^13^^,^^24^^,^^33^ While most channels have been solved in one functional state, GluCl, GlyR, and GLIC have been solved in multiple states. However, GluCl was solved in O and C states with the use of ivermectin to trap the channel in its O form, likely impacting structure.^31^^,^^32^ Similarly, GlyR has been solved in O and C forms in the presence of ivermectin or the inhibitor strychnine.^27^^,^^28^ The benefit of using GLIC (Figure 1A) is that is has available structures in O and C states (Figures 1B and 1C) obtained under conditions that differ only by the presence of the agonist (protons). As a close homolog of eukaryotic Cys-loop pLGICs, GLIC has been extensively used as a model.^34^^,^^35^ The structural details of the conformational changes for GLIC have been well-described by X-ray crystallography^24^ and simulations.^22^ In addition to pore opening/closing, illustrated in Figure 1B, changes within the bundles of transmembrane helices are evident, including the relationship between pore-forming helices M2 and M1^(−)^ (on the preceding subunit) (Figure 1C), changes to the M2-M3 loop, and changes in nearby ECD loops (*β*_1_-*β*_2_ loop and Cys loop), and critical breakage of the R192-D32 salt bridge (connecting the top of M1 and the *β*_1_-*β*_2_ loop; Figure 1A), each of which could impact anesthetic binding. Importantly, high-resolution structures have also revealed atomic-level details that allow us to locate key residues associated with different anesthetic binding sites.^14^^,^^36^^,^^37^^,^^38^^,^^39^^,^^40^

One of the most selective anesthetics is the intravenously administered propofol, which primarily potentiates inhibitory pLGICs (such as GABA_A_R and GlyR) while inhibiting excitatory Na^+^-selective channels (such as nAChR and GLIC),^5^^,^^41^^,^^42^ reducing neuronal excitation. Mutagenesis and labeling with reactive derivatives of propofol and etomidate have been used to imply an anesthetic binding site in GABA_A_R^6^^,^^7^^,^^43^ involving residues near the upper/extra-cellular ends of the M1-M3 helices, proposed to form an inter-subunit cavity,^6^^,^^7^^,^^43^ although there is evidence for sites at different subunit interfaces in this hetero-pentameric channel.^44^^,^^45^ While binding to this site in inhibitory Cl^−^ channels leads to potentiation, it is interesting that the degree and even direction of modulation via this inter-subunit site can be altered by mutations. For example, a homomeric GluCl channel has been shown to be normally inhibited by propofol via the inter-subunit cavity^46^ but can be mutated to either eliminate propofol sensitivity or to cause potentiation.^46^ This suggests that perturbations to the inter-subunit pocket can lead to a potentiating anesthetic site in distantly related pLGICs.

In the GLIC channel, this “Inter” site is nonexistent within the wild-type channel, but mutants with an enlarged inter-subunit cavity, notably F14′A and F14′A + N15′A, have been shown to bind bromoethanol and bromoform anesthetics within this cavity.^36^ The site is situated between neighboring subunits, located toward the upper portion of the TMD, with helices M1^(−)^ and M2^(−)^ in the anticlockwise direction labeled (−) and helices M2^(+)^ and M3^(+)^ in the clockwise direction labeled (+) (Figure 1D; red circle). The substitution of the bulky phenylalanine side chain (F238) with alanine^36^^,^^47^ allows anesthetic binding,^47^ while the second mutation, N239A, further enlarges the space and increases its hydrophobicity.^36^^,^^38^^,^^48^ Binding to the Inter site has been shown to exhibit stabilization of the O state in the presence of ethanol or bromoform,^36^ with propofol bridging more contacts between subunits in the O state,^38^ together suggesting a potentiating role at this site. Functionally, the mutation has been shown to shift propofol inhibition to potentiation,^36^ mimicking distantly related modulation of GABA_A_R and GlyR. It is for this reason that GLIC and its mutants offer a useful prototype for understanding anesthetic-receptor interactions in pLGICs.

GABA_A_R and GluCl structures essentially lack an intra-subunit cavity, yet structures of wild-type GLIC with desflurane or propofol revealed binding at overlapping positions within an intra-subunit site,^14^ supported by labeling with a propofol derivative. This “Intra” site is situated in the upper portion of the channel TMD between the helices of each subunit (Figure 1D; blue circle), with key residues located on helices M1, M2, and M3. Interestingly, propofol was only observed bound to GLIC’s intra-subunit region within the O state, and the site is remodeled within the C (and locally closed LC) states,^24^^,^^49^ suggesting reduced binding in the nonconducting states, supported by thiosulfonate-labeling studies.^50^ Moreover, strong concentration-dependent propofol potentiation for an Intra-stabilized mutant (M205W) channel has been observed.^38^^,^^51^ Together, this tells us that the Intra site in GLIC is also likely potentiating.

In the background of GLIC’s two reportedly potentiating TMD sites (inter- and intra-subunit), its overall inhibition^38^^,^^52^ is thought to arise primarily from its binding to the pore region.^38^ The GLIC “Pore” site has been proposed to be located near the center of the TMD in the X-ray structure, where the M2 helices of each subunit converge.^33^^,^^45^^,^^53^^,^^54^ Interestingly, studies have shown favorable binding of two anesthetic (isoflurane) molecules within the pore with high affinity, seemingly sufficient to block ion permeation as a mechanism of inhibition.^53^^,^^55^ There is therefore support for a bimodal mechanism of modulation of GLIC, with countering potentiation and inhibition.

High-resolution structures of different pLGIC channels have enabled a range of simulation studies that have aimed to examine general anesthetic binding.^39^^,^^47^^,^^51^^,^^53^^,^^55^^,^^56^^,^^57^^,^^58^^,^^59^^,^^60^^,^^61^^,^^62^^,^^63^^,^^64^^,^^65^^,^^66^^,^^67^^,^^68^^,^^69^ In terms of the GLIC channel, simulations have shed light on pathways and binding from the membrane to TMD sites, blocking of the pore, and the effects of binding on protein structure and dynamics.^39^^,^^47^^,^^53^^,^^62^^,^^63^^,^^64^^,^^65^^,^^66^^,^^67^^,^^68^^,^^70^ To better understand GLIC modulation, here, we carry out extensive MD simulations and enhanced sampling approaches on F14′A + N15′A GLIC to address key questions. Firstly, although generally indicated through X-ray crystallography, where do propofol molecules bind, and which protein residues interact most strongly to cause that binding? Describing these interactions allows for predictions for mutants to be tested experimentally. Next, what are the pathways leading from solution or the membrane to those sites within the protein? How does propofol binding alter local structure, leading to a possible induced-fit mechanism of modulation? With what affinity does the molecule bind to each site, and what is the dependence of these affinities on the channel’s conformational state? If the molecule binds with greater affinity in one state or the other, it will suggest allosteric modulation via thermodynamic stabilization corresponding to potentiation (O favored over C state) or inhibition (C favored over O state). Finally, how do binding locations and affinities depend on binding of multiple anesthetic molecules, as may occur at higher concentrations? Understanding the causes of such concentration dependence would help us understand the contributions of potentiating and inhibitory sites within a likely bimodal general anesthetic modulation mechanism for pLGICs.

We will begin with unbiased MD simulation of the protein in the presence of a high concentration of propofol to explore the possible sites. This “flooding” approach has shown success previously in finding binding sites,^62^^,^^63^^,^^71^^,^^72^^,^^73^ but it is inherently qualitative (or perhaps semi-quantitative) due to the accumulation of ligand molecules and is not the most reliable method for studying binding processes that may take a long time compared with the typical MD simulation. What is needed is an approach that reproducibly captures the inter-conversion of bound and dissociated states. For this, we invoke a multi-walker well-tempered metadynamics (MTD)^74^^,^^75^^,^^76^ approach that applies an adaptive bias to enforce the sampling of the space around and within the protein. We also apply free energy perturbation (FEP)^77^^,^^78^^,^^79^ to compare relative free energies of each site to MTD.

Using these enhanced-sampling MD simulation methods, we will comment on the possible allosteric and induced-fit mechanisms of anesthetic action. These observations offer molecular explanations for the effects of propofol that are likely to apply to other pLGICs, assisting in the development of safer anesthetics in the future.

## Materials and methods

### System building and molecular dynamics

MD simulations were conducted on the GLIC protein using the open (PDB: 4HYI^33^) and closed (PDB: 4NPQ^24^) X-ray structures. The double-mutant GLIC F238′A/N239′A was created to accommodate the propofol molecule within the inter-subunit interface,^50^^,^^80^ in addition to its intra-subunit and pore cavities. All simulations were carried out at pH 4.6 to observe propofol binding at a fixed pH, allowing for comparison to experimental measures of modulation. Experimentally, this has been done at pH 5.5 where the channel is 10% active,^38^ whereas in this study, we operate at pH 4.6, where the channel is maximally activated, and protonation states for titratable residues are more certain. These protonation states were based on our previous MD studies of GLIC.^22^^,^^81^ At pH 4.6, GLIC residues E26, E35, E67, E75, E82, E177, and E243 were protonated, while H277 and H127 were deprotonated.

Systems were constructed using the CHARMM software^99^ with specific details of each system described for each simulation type below; with one example illustrated in Figure 1a. GLIC structures were embedded in palmitoyloleoyl-phosphatidylcholine (POPC) lipid bilayers solvated with NaCl aqueous solution, with numbers of positively and negatively charged ions chosen to neutralize the net charge (+20*e*) on the protein at pH 4.6. NAMD2.12/2.13 was used for all simulations,^87^^,^^88^ which employed the CHARMM36 force field for protein,^100^ lipids,^101^ TIP3P water,^102^ and ions.^103^ Propofol was parameterized using CHARMM GENFF^104^^,^^105^ and evaluated with free energy calculations of membrane partitioning (see below). We employed a 2 fs time step, using a leapfrog integrator, together with a Nose-Hoover thermostat^106^ for temperature and Langevin piston method^107^ for pressure coupling to maintain an *NPT* ensemble with 1 atm semi-isotropic coupling in orthorhombic periodic boundary conditions. Bonds to H atoms were constrained using the SHAKE algorithm.^108^ Electrostatic interactions were calculated using particle-mesh Ewald (PME)^109^ with a coupling parameter of 0.34 and a 6th order spline for mesh interpolation. Non-bond pair lists were maintained out to 16 Å, and a real space cut-off of 12 Å (with switching distance of 10 Å) was used via an atom-based energy shift algorithm for electrostatics and force switching for Lennard-Jones.^110^ Simulation temperatures and times are specified below for each simulation type. VMD^89^ was used for analysis and visualization.

### Umbrella sampling of propofol crossing lipid bilayers

**Figure 2:**
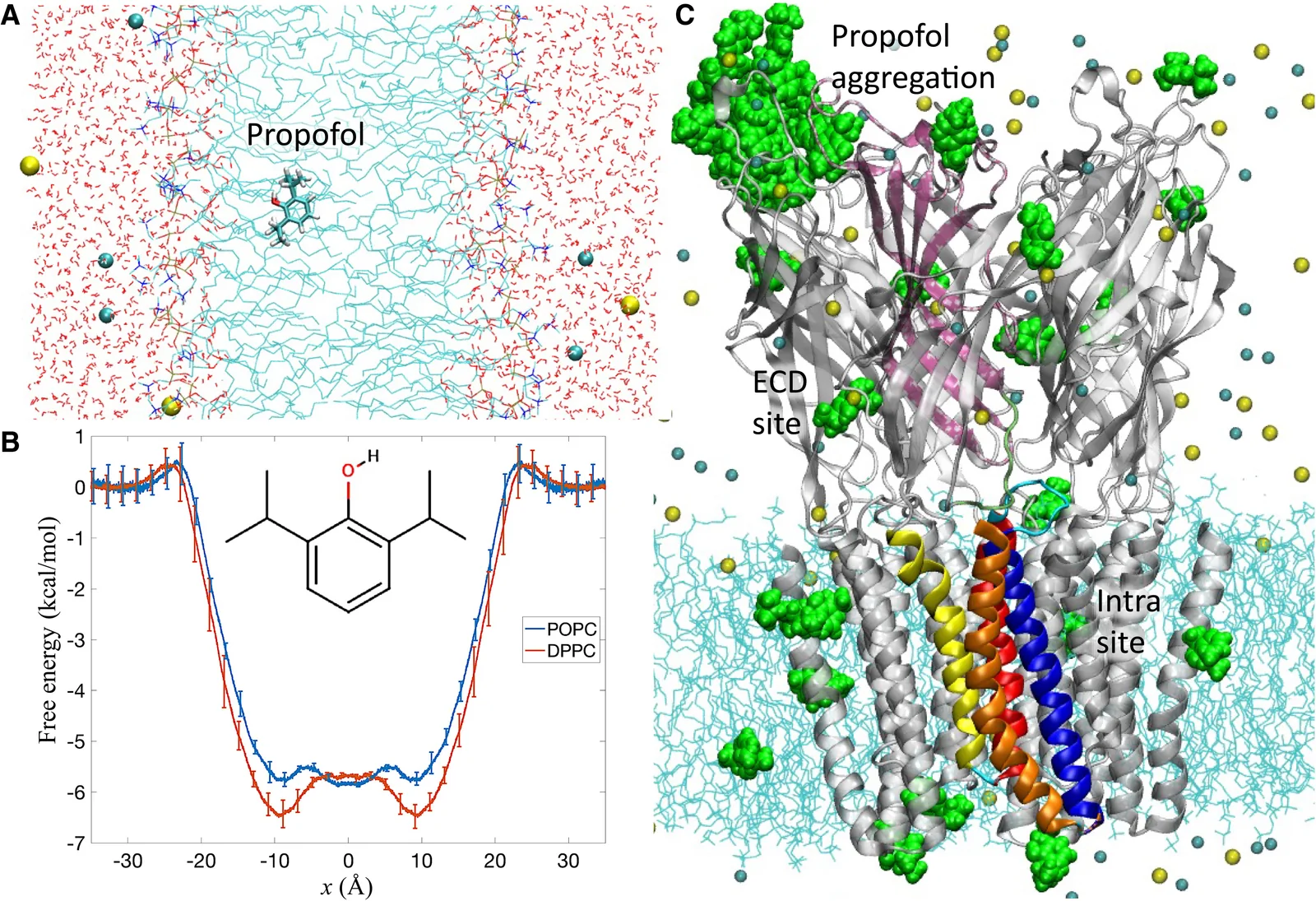
Lipid bilayer partitioning of propofol and GLIC flooding simulations. (A) Membrane partitioning system. Propofol (CPK sticks) is visualized dissolved into POPC lipids (light blue chains), with water (red dots) and NaCl ions (cyan and yellow balls) present on either side of the membrane. (B) Free energy profiles for propofol passing through POPC and DPPC bilayers. See materials and methods for discussion and Figure S1 for convergence. Error bars represent asymmetry in the raw free energy profiles before symmetrization. The propofol chemical structure is overlaid. (C) Representative structure from propofol flooding simulations of the O state, revealing aggregated propofol (green balls) adsorbed to the ECD and occupied ECD and Intra sites.

To evaluate the propofol CHARMM GENFF parameterization and to find the lipid/water partition coefficients to compare to experiments, umbrella sampling (US) was employed.^82^ Two systems were used: one with dipalmitoyl-phosphatidylcholine (DPPC) lipids to match available isothermal titration calorimetry (ITC) experiments,^83^ and 1-palmitoyl-2-oleoyl-sn-glycero-3-phosphatidylcholine (POPC) lipids to match our GLIC-MD simulation system, as used experimentally^84^ (although membrane partitioning for POPC is not available experimentally, to our knowledge). Both systems included 150 mM NaCl, with the DPPC system containing 26 and POPC 37 ion pairs. The DPPC system included 7,468 and the POPC 7,533 TIP3P molecules. After a 1-ns equilibration period, production simulations were run for each of the 71 US windows spanning ±35 Å from the membrane center, which were run for 25 ns/window. Propofol was restrained at each position using a harmonic constraint of 2.4 kcal/mol/Å^2^ (RMS fluctuation σ = 0.5 Å according to equipartition theorem), providing good overlap within the simulation time frame to converge the free energy profiles. To maintain a liquid-disordered state for the DPPC and POPC membranes, simulations were performed at temperatures of 323.15 K and 303.15 K, respectively. The windows were combined using the Weighted Histogram Analysis Method (WHAM)^85^ to produce a free energy profile, *G*(*z*), offset to be zero in bulk water. The evolution of the US simulations over time is depicted in Figure S1, showing convergence to ∼0.1 kcal/mol, yet with up to ∼1 kcal/mol asymmetry across the bilayer. The profiles were symmetrized by averaging biased density across the bilayer center, with asymmetry used as the measure of error. The partition coefficient between water and each bilayer was calculated via

$$P=\frac{1}{L}\int_{-L/2}^{L/2}e^{-G\left(z\right)/k_{B}T}dz\text{,}$$

where *L* = 50 Å. The corresponding effective free energy of binding is given by Δ*G*_eff_ = −*k*_*B*_*TlnP*. The free energy profiles (Figure 2B; presented here within materials and methods as model validation) show significant partitioning to both DPPC and POPC bilayers, with minimum binding free energies of –6.6 ± 0.2 kcal/mol at *z* ≈ 9 Å for DPPC and −5.8 ± 0.2 kcal/mol at *z* ≈ 9.5 Å for POPC. In POPC, the global minimum sits just below the glycerol region, consistent with past simulations in DPPC,^83^ which corresponds to approximately the same position as the GLIC intra-subunit binding site. This observation may help explain the affinity of lipophilic drugs for pLGIC TMD domains.^83^^,^^86^ The ΔG_eff_ values were −5.4 ± 0.1 kcal/mol for DPPC and −4.9 ± 0.2 kcal/mol for POPC, corresponding to log_10_(P) values of 3.7 ± 0.1 for DPPC and 3.5 ± 0.1 for POPC, for simulation temperatures of 323.15 K and 303.15 K (DPPC and POPC, respectively). At 323.15 K, ITC experiments using DPPC bilayers yield log_10_(P) = 5.0 ± 0.1, which corresponds to ΔG = −7.3 ± 0.1 kcal/mol.^83^ This suggests that the MD model has slightly underestimated lipid partitioning. There are no experimental data for propofol in POPC, but a previous MD study has estimated a global minimum of −4.1 kcal/mol between water and the POPC glycerol region,^86^ being slightly less negative than our result.

### Unbiased propofol-GLIC exploration: Flooding

The F238′A/N239′A double-mutant GLIC was embedded in a POPC lipid bilayer (containing 342 lipid molecules) solvated with 150 mM NaCl solution (126 Na^+^ and 146 Cl^−^) at pH 4.6. Systems contained 45,670 (O state) or 46,134 (C state) water molecules, with a total of ∼210,000 atoms each. For the O state system, 50 mM of propofol (42 molecules) was distributed randomly within the bulk aqueous regions. Following observations of complete propofol partitioning to the membrane in the O state simulation, the C state simulation commenced with propofol distributed within the POPC membrane (to avoid a slow initial equilibration period). Both systems were simulated at 303.15 K for 3.4 and 3.1 μs, respectively. Only one simulation for each conformation was run because of the limited success of the unbiased/flooding MD approach. The free energy surface for binding (Δ*G*(*x*,*y*,*z*)) of propofol was calculated using the average density, *ρ*, as function of *x*, *y*, and *z* collective variables (CVs), as defined below, using

$$\Delta G\left(x,y,z\right)=-k_{B}T\;\ln\;\rho (x,y,z),$$

Because both O- and C-state channels were simulated at pH 4.6, conformations were maintained by biasing the distance between the M2 and M1^(−)^ helices to control the state of the GLIC TMD pore^22^ (small value corresponding to M2 being held away from the pore axis in an O state). This CV was defined as the distance between the center of mass of the C_α_ atoms of residues L241 to N245 on the M2 helix and the center of mass of the C_α_ atoms of residues I200 to P204 on M1^(−)^. To ensure preservation of the O conformation, this CV was prevented from exceeding a separation distance of 14.5 Å using a flat-bottom constraint with steep force constant 20 kcal/mol/Å^2^ (based on the clear separation of O and C/LC states associated with that value previously^22^). For the C state, the same constraint ensured the M2-M1^(−)^ distance remained over 14.5 Å. These constraints effectively maintained the desired channel conformations while allowing for unperturbed dynamics within the specified range. Although the position of these restraints was chosen to have minimal influence on O- and C-state dynamics, they may impact propofol binding to some degree, in particular in inter-subunit sites that reside close to the M2 and M1^(−)^ helices (Figure 1D).

### Metadynamics of single-propofol binding

Metadynamics (MTD)^74^ was employed using well-tempered (WT-MTD)^75^ and multi-walker extensions (MW-MTD).^76^ Each MTD replica system consisted of the GLIC protein embedded in a membrane with 243 POPC lipids, hydrated by 30,647 water molecules and 146 Na^+^ and 166 Cl^−^ ions (∼260 mM), totaling 150,320 atoms. The system had 40 replicas and a well-tempering bias temperature,^75^ Δ*T*, of 1,000 K, approximately three times the system thermostat temperature of 330 K (with 330 K chosen to slightly accelerate dynamics). That is, the WT-MTD used a bias factor $\gamma =\frac{T+\Delta T}{T}=4.03$. This bias temperature was chosen to ensure higher accuracy and convergence, despite slower exploration. The initial hill weight was 0.05 kcal/mol, with hill width 1.25 Å. The O-state MTD explored an 80 Å × 80 Å x 90 Å grid centered on the TMD pore, while the C-state MTD explored an 80 Å × 80 Å x 80 Å grid. Gaussians were deposited every 1 ps on the *x-y-z* grid. A 5 kcal/mol/Å^2^ flat-bottom constraint was applied 5 Å from the sides of the grid to prevent propofol from escaping. The same flat-bottom restraint on M2-M1^(−)^ distance as used in unbiased MD simulations above was used to maintain O and C conformations during MTD.

The relative positions of propofol and GLIC were determined using three orthogonal vectors as CVs representing the *x*, *y*, and *z* directions. The CV for the *z* direction was defined by an approximate channel axis vector that was formed from the centers of mass of two sets of atoms in the TMD. The lower set included backbone atoms from residues 274 to 276 on the M3 helix and 293 to 295 on the M4 helix. The upper set included backbone atoms from residues 198 to 200 on M1, 239 to 241 on M2, and 254 to 256 on M3. Each subunit had these selections. The *x* CV selections were made by aligning the pore and one of the M4 helices along the *y* axis of the system, with the backbone atoms of the TMD on either side of the axis chosen as the two selections to define the vector. Likewise, defining the *y* CV required aligning the protein over the *x* axis. With these definitions, angles between *x*, *y*, and *z* vectors were maintained ∼90° to within ∼±4°, with the origin within ∼1 Å from the pore center. Results are reported with an offset to ensure the pore center is at the origin.

Unlike the unbiased simulations, each MTD replica contained a single propofol. The initial position of the propofol in each replica used a membrane position taken from the previous MD simulations to reside next to one subunit of the TMD. The duration was 815 ns/replica for the O state and 741 ns for the C state, equivalent to 35.0 and 30.8 μs, respectively. Simulation length was determined by the time needed for the binding sites in each GLIC subunit to be accessed and free energy map converged, as illustrated in Video S1. The simulations were extended until propofol visited all subunits, resulting in visually distinguishable sites and pathways in the free energy surface. To effectively compare the O- and C-state PMFs, a common reference far from the channel within the membrane was chosen. This involved a cylindrical region centered around *x* and *y* coordinates [–23.5, 23.5] with a radius of 4 Å. This region encompassed the POPC bilayer with *z* between ±20.5 Å. While convergence was pushed to within a fraction of a kcal/mol (Figure S3), small differences between the free energy surfaces for the five subunits remain. To symmetrize, the surface was rotated in 72° increments around the *z* axis and Boltzmann averaged.

### Metadynamics with propofol cooperativity in inter-subunit sites

To explore the impact of anesthetic binding in multiple GLIC subunits simultaneously, additional MTD simulations were run where sampling focused in the region of one subunit, while propofol molecules were bound to the neighboring subunits. The MTD setup remained unchanged, except for the addition of extra propofol molecules, permanently held within each of four other subunits, within the Inter sites (between residues N200, A238, and E243). The MTD simulation utilized the same CV grid as above, but with adjusted boundaries (60 Å × 60 Å x 125 Å) to focus on the one subunit that does not have a permanently bound propofol, as well as the pore and immediately surrounding membrane. The expanded *z* range of the grid was chosen to capture binding near the TMD-ECD interface. To confine propofol within the designated region, a flat-bottom constraint of 30 kcal/mol/Å^2^ was applied, creating a wall 1 Å from the edge of the MTD box. Additionally, a 0.44 kcal/mol/Å^2^ harmonic RMSD constraint was used to weakly anchor the 4 propofol molecules in the other Inter sites. MTD was initially run with 16 walkers/replicas, each positioned at one of the sites within the TMD. To enhance sampling beyond the TMD, the number of walkers/replicas was increased to 40, derived by branching off from different frames of the initial replica trajectories. Each initial replica in the O state had a runtime of approximately 140 ns, while in the C state, each replica ran for approximately 280 ns. The branched replicas had run times of 65 ns and 34 ns, respectively. Overall, the total simulation time for these additional multi-propofol calculations was 3.8 μs in the O state and 5.3 μs in the C state.

### Free energy maps and analysis

The free energy surface for propofol binding, Δ*G*(*x*,*y*,*z*), was obtained from the inverse bias of the multi-walker MTD.^77^ This 3D surface was offset such that the average over the distant membrane region (defined above) was set to zero. Specific 2D projections have been created using Boltzmann integration to allow for visualization. For example, to obtain a projection of the probability as function of *x* and *y* variables, one integrates the joint probability that is a function of *x*, *y* and *z* over a defined range of the third coordinate *z*. In terms of free energy, this involves

$$\Delta G\left(x,y\right)=-k_{B}T\;\ln\;\int_{z_{\min}}^{z_{\max}}e^{-\Delta G\left(x,y,z\right)/k_{B}T}dz+C\text{,}$$

with the additive constant *C* determined by setting the free energy in a 2D region considered to represent the distant membrane (defined above) to zero. Projections of the TMD in the *xy* plane used a *z* range from −0.5 to 19.5 Å, capturing relevant sites and pathways. For *xy* projections of the ECD from unbiased simulations, a *z* range of 24.5–89.5 Å was used to highlight an observed ECD binding site. Similarly, the GLIC ECD was visualized in the *xz* plane by summing over the *y* range −14.5 to 14.5 Å. In the O state, the TMD Intra and Inter site minima in one subunit were visualized in the *xz* plane using a *y* range of 5.5–11.5 Å. Due to shifts in the GLIC M2 helices between states, Intra and Inter sites did not align along a single axis in the C state. The C state Intra site was visualized using an *xz* projection with *y* spanning 10.5 to 12.5 Å, while the Inter site used 13.5 to 14.5 Å. The Pore site involved *xz* projections with *y* spanning −9.5 to 10.5 Å. To visualize the pathway between the Intra and Inter sites, the free energy map was rotated around the pore by 37° clockwise. After rotation, the pathway was visualized using an *xz* projection over *y* range 9.5–16.5 Å (O state) or 14.5 to 18.5 Å (C state).

To obtain a binding free energy for a given binding site, relative to the membrane, the full 3D Potential of Mean Force (PMF) (already offset relative to the distant membrane; see above) was integrated according to

$$\Delta G_{site}=-k_{B}T\;\ln\;\int_{site}e^{-\Delta G\left(x,y,z\right)/k_{B}T}dxdydz,$$

where the site was determined by all of the grid points with centers that lay within a sphere of radius 2.0 Å around the center of the minimum (except for the pore site, which was instead determined by a 4-Å-radius cylinder spanning −10 < *z* < 15 Å). To obtain measures of statistical error in the free energies, the 3D PMF was analyzed by rotating around the channel axis by multiples of 72° to obtain 5 separate measures, leading to one standard error of means based on 5 measurements for each grid point. These errors were then propagated for the above Boltzmann integrals via a Boltzmann-averaged error. That is, for a set free energies for grid point *i*, Δ*G*_*i*_, with errors *δ*Δ*G*_*i*_, the error in the overall free energy, $\Delta G=-k_{B}T\;\ln\;\sum_{i}e^{-\Delta G_{i}/k_{B}T}$, is found via the total derivative of Δ*G*, leading to

$$\delta \Delta G={\left(\sum_{j}e^{-\Delta G_{j}/k_{B}T}\right)}^{-1}\sum_{i}e^{-\Delta G_{i}/k_{B}T}\;\delta \Delta G_{i}.$$

This propagation was used to obtain the 2D error map shown in Figure S4, as well as to obtain the error estimate for each binding site from MTD. Elevated errors at a distance from the TMD center can arise due to variations in site locations around the pentamer, which are not exactly 5-fold symmetric at every MD step, despite similar sampling of each local minimum within each subunit. For the Pore site, 5-fold symmetric sites do not exist, such that the error in the region was instead calculated with time-based block averaging (based on 5 samples) and standard error of means.

### Interaction analysis

To determine the enthalpic contributions of different protein residues to the stabilization of propofol in each site, mean interaction energies were computed using the NAMDenergy tool.^87^^,^^88^^,^^89^ The contribution from propofol-water interaction energy was also calculated, with water molecules within 10 Å of propofol selected. To calculate errors, the data set was divided into 5 blocks, and the standard error of the mean was calculated. Additional contact probability analysis for the propofol hydroxyl group (O or H atom) related to hydrogen bonding to protein (O, N, or polar H atoms) or water (O or H atoms) has been computed, with relevant cutoff distances (determined from analysis of radial distribution functions; not shown) specified in Table S2.

### Free energy perturbation

Alchemical free energy perturbation^78^^,^^79^ (FEP) has been used to calculate the free energy of propofol binding to each site (relative to the membrane) within the O or C states of the F238A/N239A GLIC channel at pH 4.6. This included Intra1, Intra2, Inter1, Inter2, and Pore sites. FEP simulations were performed for each site in both the O and C conformations of GLIC. In the C-state PMF, an additional “Inter3” site between Intra and Inter sites was also studied. Each FEP sampled its site using a spherical flat-bottom constraint with 3-Å radius. All flat-bottom constraints used a force constant of 30 kcal/mol/Å^2^. The center of each flat-bottom constraint was determined using the free energy map obtained from the multi-propofol MTD simulation, applied relative to the C_α_ of surrounding residues (within ∼5 Å of the center; see Table S1). For the Pore site, a cylindrical flat-bottom constraint aligned with the *z* CV was used. The cylinder had a 4-Å radius with planar flat-bottom constraints at *z* = −10 and 15 Å to trap inside the pore.

For the membrane reference, a propofol molecule was inserted at lab-frame position [–37.4, 38.8, 21] Å. A 1.6 kcal/mol/Å^2^ harmonic constraint (σ = 0.5 Å) was applied to confine it to the *xy* position. In the *z* direction, the molecule was limited to 6–12 Å from the center of the membrane with flat-bottom planar potential, encompassing the glycerol region where the propofol preferentially binds. To anchor GLIC within the system, the protein was weakly bound with a 1 kcal/mol/Å^2^ harmonic constraint near lab-frame coordinate [6.0, −4.8, −17.7] Å (O state) or [5.0, −5.0, −18] Å (C state).

FEP simulations used a dual-topology method,^90^ with soft-core potentials to facilitate particle annihilation and creation (soft-core van der Waals radius-shifting coefficient of 0.6, with default ranges for electrostatics and van der Waals switching used).^88^ Simulations were run at 330 K (to match the MTD) and began with a series of 1-ns “pulling” simulations, incrementally changing the coupling constant, λ, from 0 to 1 and back to 0. This produced 10 endpoints in each direction, creating 20 windows. Each window was simulated in parallel for 12–16 ns (totaling 240–360 ns) to ensure convergence (Figures S9 and S11). The VMD ParseFEP plugin^91^ was used to calculate Δ*G* values using Bennett Acceptance Ratio.^92^

## Results

### Unbiased sampling of propofol binding to GLIC

Unbiased flooding simulations were used to explore propofol binding to the O- and C-state F238A/N239A GLIC structures. Propofol molecules quickly populated the membrane from aqueous solution, consistent with calculations of strong lipid bilayer partitioning (Figures 2A and 2B). Figure 2C shows a sample snapshot of propofol binding to the O-state GLIC. Figure S2 shows the corresponding free energy surfaces to these unbiased sampling simulations for both the O and C states. However, the strong membrane partitioning (Figure S2B; dark blue membrane region) led to limited sampling of the binding sites within the protein’s TMD, sampling only the O-state Intra site at the channel-membrane interface (Figures 2C and S2A), with an approximate binding free energy of −1.6 ± 0.5 kcal/mol. There was no sampling of the Inter site or the pore of the channel in either (O or C state) >3 μs simulation. Interestingly, when exploring the O state-ECD, interaction between propofol and a site within the ECD, previously suggested to bind ketamine,^63^^,^^65^ was seen between residues K183, K152, and Y23 in one subunit only (Figures S2B–S2D). We note that unexplained density was observed at this site within the X-ray crystal structure of O-state GLIC in the absence of ketamine,^13^ yet these simulations suggest that it can bind propofol at that location.

Overall, these unbiased simulations have limited ability to access the different binding sites within the 5 subunits of the GLIC TMD. We therefore turned to an accelerated sampling method for a more complete description of propofol binding.

### Metadynamics exploration of single-propofol binding to the GLIC open state

**Figure 3:**
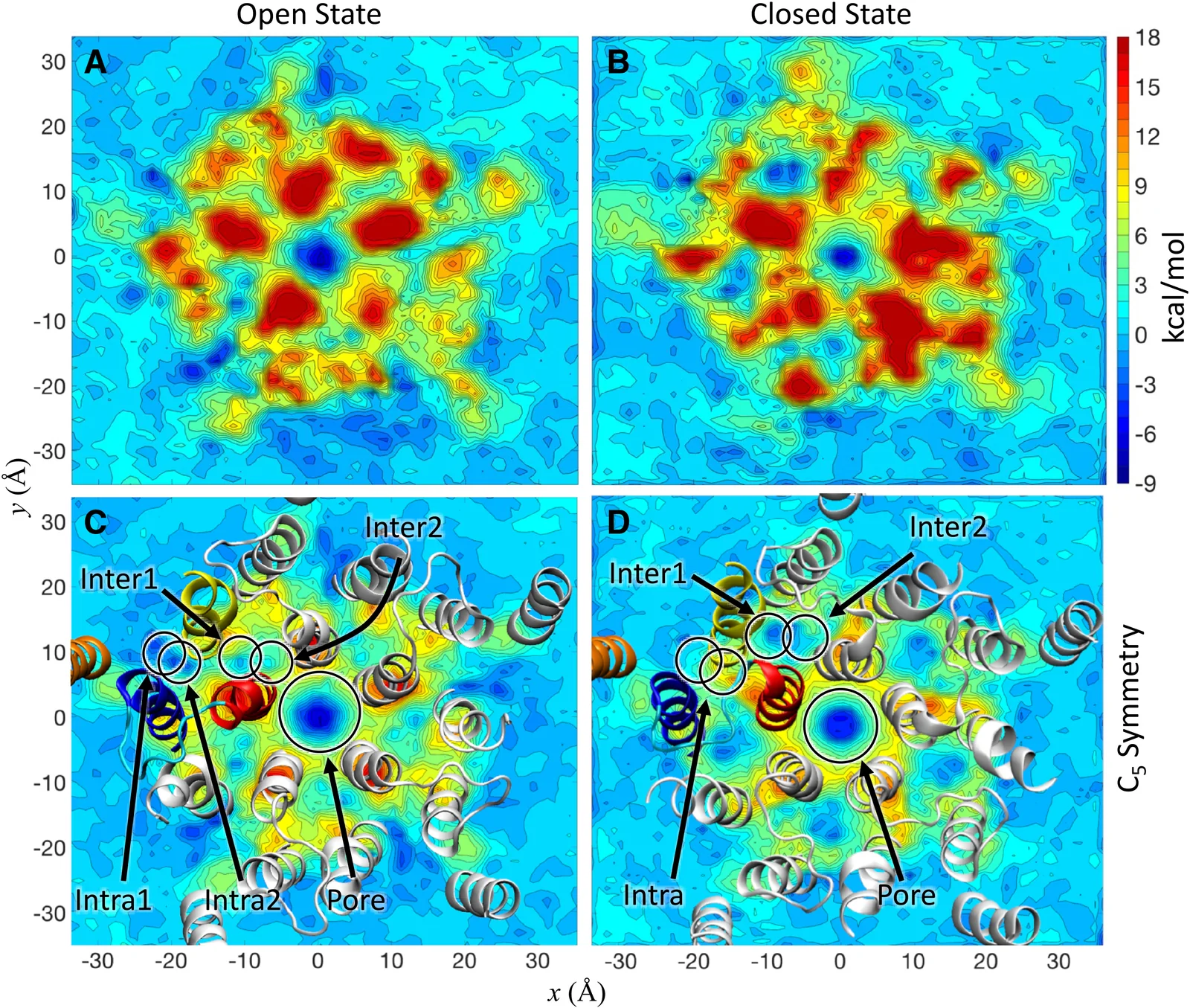
Metadynamics free energy maps for propofol binding. (A) and (B) show *xy* projections for O and C states at pH 4.6, respectively. (C) and (D) show 5-fold symmetrized maps with structures superimposed for O and C states, respectively. Each of the binding minima have been circled and labeled, including the Intra1 and Intra2 sites, the Inter1 and Inter2 sites (in the (+) subunit), and the Pore site. The reference 0 kcal/mol free energy is the average free energy in the distant membrane. Errors are shown in Figure S4.

The multi-walker well-tempered MTD simulations were seen to quickly explore the membrane and protein interface, and over time, they were able to penetrate into each subunit of the GLIC pentamer, allowing for quantitative calculations from a converged free energy map. Figure S3A shows the time evolution of the free energy for an *xy* projection in the O state; see also Video S1), revealing exploration of the entire GLIC pentamer and convergence to kcal/mol order, achieving a near 5-fold symmetric map (see Figure S4A for error analysis based on asymmetry). Figure 3A presents an *xy* free energy projection for the O state, revealing the extent of sampling of the propofol molecule across the 5 subunits of the protein. Figure 3C shows the 5-fold symmetrized results with superimposed GLIC structure, including the locations of Intra, Inter, and Pore binding sites. Moreover, the sampling between membrane and electrolyte was sufficient to estimate the ΔG_eff_ between water and membrane of −5.5 ± 0.5 kcal/mol, corresponding to a partition coefficient of *log*_*10*_(*P*) = 3.6 ± 0.3, consistent, within errors, to the US calculations for propofol crossing lipid bilayers above.

**Figure 4:**
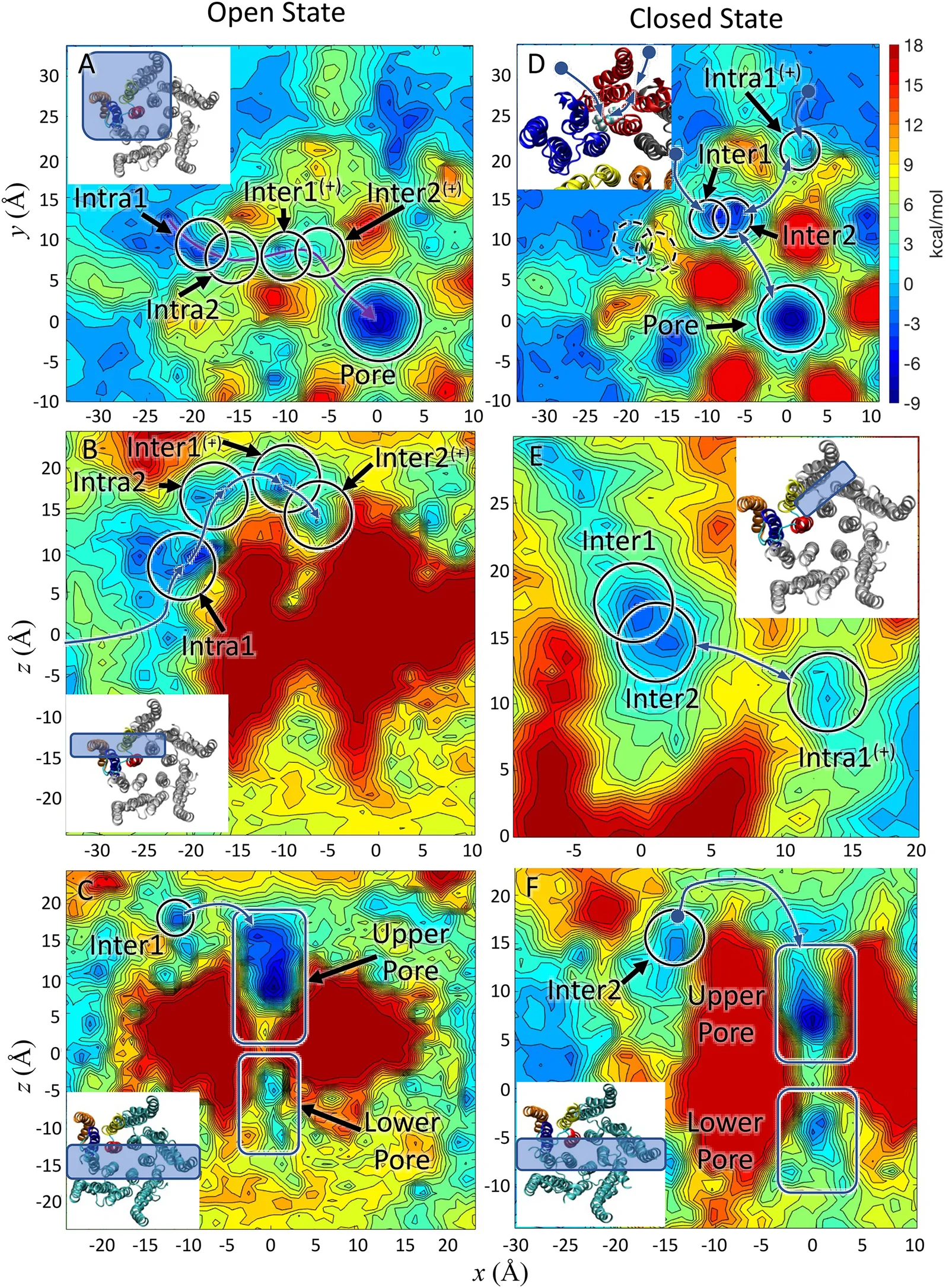
Zoomed free energy projections from MTD showing sites and pathways. (A) *xy* projection for O state; inset shows path traveled between sites. (B) *xz* projection for O state. Sites and pathways are indicated with circles and arrows. (C) Projection showing the pore site (unsymmetrized to best reveal sampling in the single central pore) for O state. (D) and (E) match (A)–(C) but are for the C state. Graphs have varying axis ranges to highlight features. Inset in (D) has TM helices colored by subunit (not by segment) to highlight the inter-subunit movement. (E) has different *x* and *y* ranges owing to the use of a rotated cross-section to improve visibility, as illustrated in the inset.

To examine the binding surface in more detail, Figure 4A provides a zoomed-in view around one subunit in this *xy* projection, with Figure 4B showing the corresponding *xz* (side-on) projection. The Intra site can be seen to contain two local minima, one further down in the TMD (Intra1) and another along the path to the Inter site (Intra2). The Inter site is also bifurcated with two local minima labeled Inter1 and Inter2. The Pore site exhibits distinct upper and lower minima. Examining the MTD trajectories, the sequence in time that propofol was observed to visit each site was noted. Propofol first entered the Intra site from the membrane interface. It initially occupied the Intra1 minimum; it then moved upward to Intra2 further into the protein. Traveling via a low free energy path (in a clockwise direction in Figures 4A and 4B), the molecule reached the Inter1^(+)^ minimum (next subunit). This site sits high in the TMD and allows propofol to access the Pore site (Figure 4C). Inter1 also directly connects to Inter2, allowing propofol to traverse between them with minimal barrier (Figure 4B). Propofol cannot access the Inter^(−)^ sites (lower part of Figure 4A) from the Intra site, as a high barrier is created by the presence of the M2 helices. Overall, the path of propofol through the O state of GLIC resembles a horseshoe-shape connecting the Intra and Inter site minima (Figure 4B).

#### Intra-subunit sites: O state

**Figure 5:**
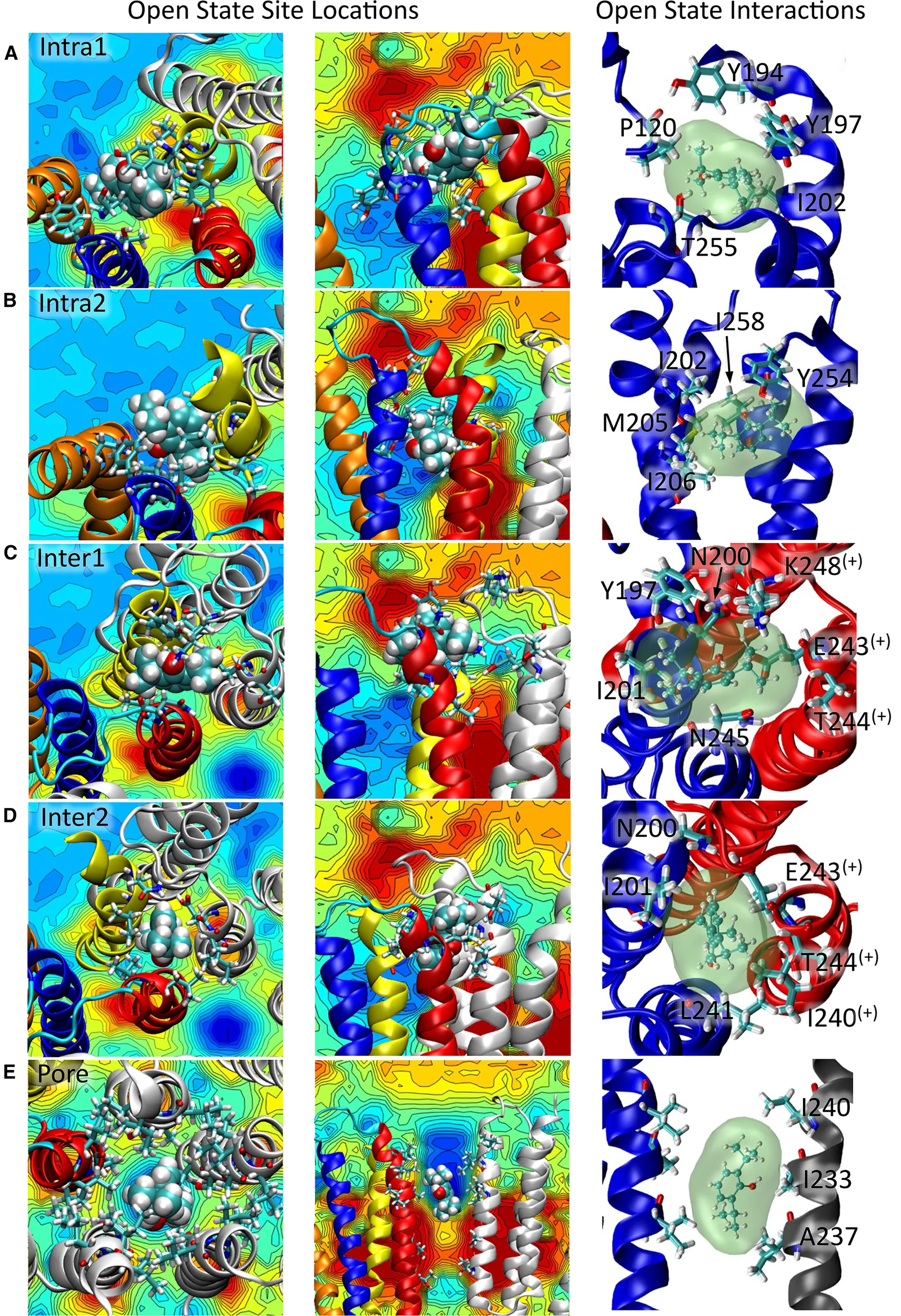
Open-state sites and interactions. Each TMD has propofol bound to a different site, along with their key residues. Figures on the left are *xy* PMF projections. Those in the middle are *xz* PMF projections. TM helices in one subunit are colored by segment in the left and middle columns. Structures in the right column show the key residues that interact within each site (with TM helices colored by subunit to highlight the intra- and inter-subunit sites). (A) Intra1; (B) Intra2; (C) Inter1; (D) Inter2; (E) Pore.

During the O-state MTD exploration, two minima were observed in the intra-subunit region (Figures 4A and 4B). Intra1, accessed first from the membrane, exhibited a free energy of −4 ± 2 kcal/mol relative to the surrounding lipid. Intra2 had a higher free energy of 0 ± 2 kcal/mol. Intra1 interacted most strongly with residues I202, I258, and I206, as seen in Figure S5A and discussed in the supplemental information. The Intra2 site was dominated by T255, Y197, and I202 (Figure S6B). Important residues are visualized in Figures 5A and 5B.

Experimental evidence derived from X-ray crystallography and mutagenesis points toward binding in the GLIC Intra site being dominated by residues M205, V242, and T255(39). T255 exhibits interactions with propofol in both Intra1 and Intra2, being slightly stronger within Intra2 (Figures S6A and S6B). Residue V242 contributes to Intra1, while M205 contributes to Intra2. The importance of M205 is highlighted by the experimental choice of its mutation to Trp to stabilize the Intra-site propofol and enhance potentiation.^38^ The strong interactions of T255 and I202 residues in both locations indicate a role in the interconnectedness of the two minima. Interestingly, propofol’s phenol group interacted with T255 only around 10% of the time while in the Intra1 site, and not at all in the Intra2 site (Table S2). The mean interaction energy of propofol with water in the O state was greater in Intra1 (Figure S6E), which may be attributed to its exposure to electrolyte at the protein periphery. In Intra1, water formed hydrogen bonds with propofol 17% ± 7% of the time, and not at all in Intra2 (Table S2).

#### Inter-subunit sites: O state

Each simulation revealed two distinct inter-subunit minima: Inter1 and Inter2 (Figures 4B and 4C). Propofol enters the Inter1 site by traveling along a pathway between Intra2 and Inter1^(+)^ with a barrier of +2 ± 2 kcal/mol. Within Inter1, it has a free energy of −1 ± 2 kcal/mol, while in Inter2, it is elevated at +1.9 ± 0.9 kcal/mol. The centers of these minima were separated by 6 Å with a barrier of 3 ± 2 kcal/mol connecting them. We note that propofol is ∼10 Å long, and thus, two molecules are not likely to bind in these two minima at the same time.

Important residues in each Inter site are visualized in Figures 5C and 5D. Residue interactions in the Inter site were analyzed separately for the anticlockwise (−) and clockwise (+) sides. Propofol in Inter1 was strongly bound to N245^(−)^, Y197^(−)^, and I201^(−)^. The (+) side was dominated by E243^(+)^ and K248^(+)^ (Figures 5C and S6C). Inter2 shared key residues with Inter1 and was defined by N200^(−)^, L241^(−)^, and I201^(−)^. The opposing side featured E243^(+)^ and I240^(+)^ (Figures 5D and S6D). X-ray crystallography studies have identified residues N200^(−)^, A238^(−)^, A239^(+)^, and E243^(+)^ as key contributors to the Inter site.^38^ MTD simulations in this study support N200 and E243 but revealed only weak interactions with the mutated A238 and A239 residues, as may be expected. In Inter1, the propofol hydroxyl group coordinated with residues N200, Y119, N245, V242, and I201. Similarly, Inter2 exhibited interactions with residues N200, I201, T255, L241, and I240 (Table 1). Although propofol had interactions with multiple residues in both sites, only N200 and I201 were consistently identified as crucial for Inter site binding. Water plays a greater role in the Inter sites compared with the Intra sites. The mean contributions from water were similarly high in each Inter site (Figure S6E). Inter1 is located near the top of the TMD where it is exposed to water, more so than Inter2 below it. In Inter1, propofol formed hydrogen bonds with water molecules 29% ± 6% of the time, while in Inter2, this occurred only 15% ± 6% of the time (Table S2).

**Table 1:** Summary of binding sites, free energies, and strongly interacting residues

Site	Free energy		Important residues in each structural feature								
	*(kcal/mol)*		*β*_1_/*β*_1_-*β*_2_ loop	Cys loop	Loop F	Loop C	Pre-M1	M1	M2	M2-M3 loop	M3
	O pH4.6	C pH4.6									
	Single-propofol MTD (multi-propofol FEP^†^)		(16–35)	(112–122)	(143–162)	(173–183)	(∼192–194)	(195–217)	(222–245)	(246–253)	(254–281)
Intra1	−4 ± 2 (−0.3 ± 0.5)	+0 ± 1 (+1.1 ± 0.3)	–	P120	–	–	Y194	Y197, I202 I202, M205	V242	–	T255 Y254, T255, I258
Intra2	0 ± 2 (−7.0 ± 0.5)	+3 ± 2 (−3.0 ± 0.5)	–	–	–	–	–	I202, M205, L206	–	–	Y254, I258
Inter1	−1 ± 2 (−1.5 ± 0.2)	−4 ± 2 (−3.9 ± 0.5)	–	–	–	–	–	Y197, N200, I201 N200	V242, E243^(+)^, T244^(+)^, N245 L241, N245, E243^(+)^	K248^(+)^ M252^(+)^	G256^(+)^, I259^(+)^, F260^(+)^
Inter2	+2 ± 1 (−4.0 ± 0.4)	−3 ± 1 (−3.8 ± 0.3)	–	–	–	–	–	N200, I201 N200, P204	A238, I240^(+)^, L241, E243^(+)^, T244^(+)^ L241, E243^(+)^	–	G256^(+)^, I259^(+)^, F260^(+)^
Inter3^∗^	N/A (N/A)	N/A (+1.2 ± 0.2)	D32	Y119	–	–	R192	Y197, I201	N245	T249^(+)^, P250^(+)^	–
Pore (mid-upper)	−6.7 ± 0.7 (−9.3 ± 0.3)	−6 ± 1 (−8.3 ± 0.4)	–	–	–	–	–	–	T226, S230, I233, A237, I240 S230, I233, A237, I240	–	–
Pore (lower)	−1.0 ± 0.7 (N/A)	−2 ± 1 (N/A)	–	–	–	–	–	–	T222, T226	–	–
ECD site^∗∗^	N/A (N/A)	N/A (N/A)	Y23	–	K152	K183	–	–	–	–	–

For each site, the free energy (relative to the membrane) from single-propofol MTD simulations is specified, while FEP values from the multi-propofol simulations are provided in parentheses. Note that in the C state, the Intra2 minimum did not exist, and the free energy corresponds to the region where it was seen in the O state. Residues seen to be involved in strong interactions within each propofol binding site are listed (see Figures S5 and S6 for a full set). Residues have been grouped according to the structural features in which they belong, with their residue ranges specified. Note that pre-M1 is defined as residues ∼192–194 here, but residue 192 may be considered to lie on strand *β*_10_. ^†^ “Multi-propofol” refers to the additional MTD simulations that focused on the region around one subunit where a propofol was held within each of the other 4 subunit Inter sites (please confirm capitalization). ^∗^Site “Inter3” has been identified only within multi-propofol simulations in the C state (see Figure S10E for a full set of interactions). ^∗∗^ECD site (ketamine-like) was observed during unbiased MD simulations of Figures 2C and S2B–S2D.

#### Pore sites: O state

The Pore site reveals two distinct minima separated by a less interactive segment in the middle of the pore (Figures 4C and 5E). The free energy of the entire pore was −6.3 ± 0.7 kcal/mol, representing the most important binding site overall. Binding in the Pore site is dominated by a large upper free energy minimum (3 < *z* < 15 Å), with a free energy of −6.7 ± 0.7 kcal/mol. The lower Pore minimum (−10 < *z* < −0.5 Å) has a free energy of just −1.0 ± 0.7 kcal/mol. Important residues in the Pore are visualized in Figure 5E. The most interactive residues were I240, A237, I233, and S230 (Figure S6E), with each forming a 5-residue ring that binds propofol simultaneously. Experiments have highlighted the role of S230,^33^ but our analysis suggests greater roles are played by I240, A237, and I233. Propofol in the Pore site is well hydrated within the O state (Figure S7E), and water hydrogen bonds to the phenol group 57% ± 12% of the time (Table S2).

### Modified binding sites and pathways in the closed state

Structural differences between the O and C states (Figure 1C) impact the arrangements of residues lining the TMD binding sites, affecting the locations and pathways of binding. Figure 3B displays an *xy* projection of the C-state MTD free energy surface for propofol binding, while Figure 3D shows the symmetrized map, identifying binding sites (see Figures 4D and 4E for zoomed-in views of *xy* and *xz* projections). Figure S3 shows convergence of the free energy for an *xy* projection in the C state (see Figure S4B for error analysis). In the C state, propofol exhibits similar sites, with the exception of Intra2, which is not evident in the map. Propofol still enters the TMD through Intra1 from the membrane, but in the absence of an Intra2 site, it then moves to Inter2^(−)^ to access the Inter site (Figure 4E), being very different from the pathway seen in the O state. Access to the Pore is unchanged in the C state, being able to pass from Inter1 through a space near the top of the M2 helices into the upper Pore.

#### Intra-subunit site: C state

**Figure 6:**
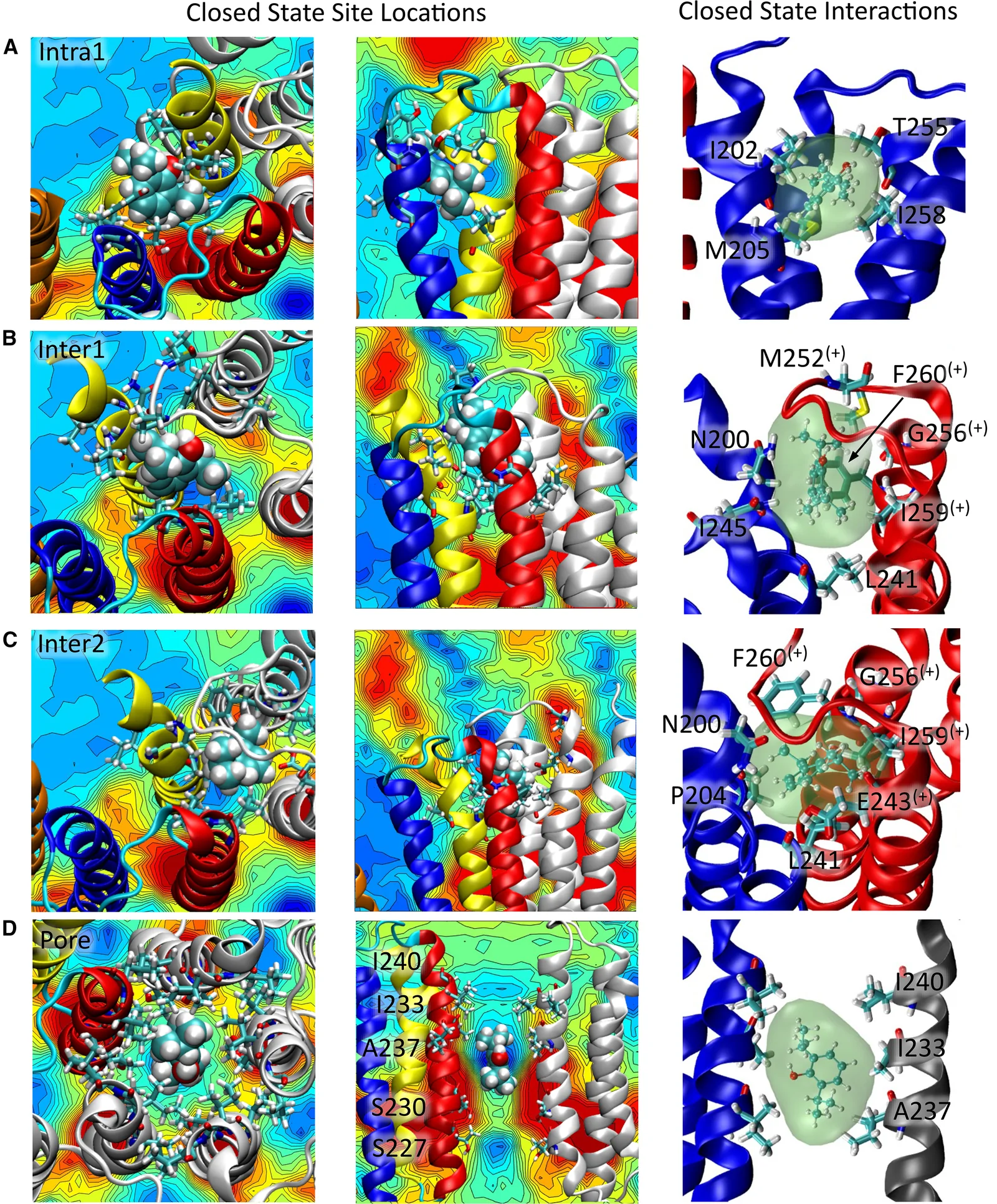
Closed-state sites and interactions. Each TMD has propofol bound to a different site, along with their key residues. Figures on the left are an *xy* PMF projection. Those in the middle are *xz* projections. TM helices in one subunit are colored by segment in the left and middle columns. Figures on the right display the key residues binding in each site. (A) Intra1; (B) Inter1; (C) Inter2; (D) Pore. Structures in the right column show the key residues that interact within each site (with TM helices colored by subunit to highlight the intra- and inter-subunit sites).

The transition between the O and C state changes the position of the M2 helices that collapses the Intra-Inter^(+)^ pathway and eliminates the Intra2 minimum. The remaining Intra1 site was found to have a free energy of 0 ± 2 kcal/mol, while the location of the previous Intra2 now corresponded to a barrier of +4 ± 2 kcal/mol (Figure 4D). Important residues in the Intra site are visualized in Figure 6A. Key interactions include I202, I258, T255, and M205 (Figure S6A). These residues are similar to those identified in the O state, except for V242, which no longer contributes. Hydrogen bonding with the propofol OH group included residues I201, I202, I206, and M205 (Table S2). Of these, I202 and M205 play important roles in Intra site binding, while I206 contributes significantly less than in the O state, where it was important in forming the (now absent) Intra2 minimum. The strength of propofol-water interactions in the C-state Intra site is reduced (Figure S6E) compared with the O state, though within error. H-bonding interactions between propofol and water in the C-state Intra1 site were observed only 2% ± 1% of the time (Table S2).

#### Inter-subunit sites: C state

The altered orientation of the M2 helix in the C state has shifted the Inter minima further in the (+) direction, affecting its relation to the Intra sites on neighboring subunits (Figure 3D versus C). The free energy of propofol in the Inter1 site is now –3.4 ± 2.0 kcal/mol, while that in the Inter2 site is now −2.9 ± 1.3 kcal/mol (Figures 3D, 4D, and 4E), being deeper than in the O state, suggesting an apparent contradiction with experimental measurements of potentiation via the Inter site,^36^ which we return to discuss below. Important residues in the Inter sites are visualized in Figures 6B and 6C. In the C state, Inter1 is defined by interactions involving N200^(−)^, L241^(−)^, and N245^(−)^ on the (−) side subunit, as well as F260^(+)^, M252^(+)^, and G256^(+)^ on the (+) subunit (Figure S6B). Inter2 is made up of N200^(−)^, L241^(−)^, P204^(−)^, and N245^(−)^ on the (−) subunit, as well as I259^(+)^, F260^(+)^, and G256^(+)^ on the (+) subunit (Figure S6C). Inter1 and Inter2 share strong interactions with N200. Mutated residues A238 and A239 again do not play a prominent role.

Because of the shift in M2 helices, exposing Inter sites more to the (+) side of the subunit, there were almost no common key residues between the O and C state Inter minima from the (+) side subunit. In the C state, there exists stronger interactions with N200 and weaker interactions involving N245 and E243 within Inter1, compared with the O state. There also exist weaker interactions with N200, L241, and I201 in the Inter2 site, compared with the O state. The only propofol hydroxyl interactions identified in Inter1 or Inter2 were N200 and I201 (Table S2), suggesting that propofol may have been restricted from rotating freely in the C state Inter sites, primarily due to a strong interaction with N200 (Figures S7B and S7C). Again, Inter1 and Inter2 minima both had strong water interactions, similar to the O state (Figure S6E). However, Inter2 is positioned higher in the TMD compared with the O state, increasing access to electrolyte, resulting in slightly stronger water interaction energy. The Inter1 and Inter2 minima involved propofol H-bonds with a water molecule in 38% ± 9% and 29 ± 18% of instances (Table S2), being elevated in Inter2 compared with the O state.

#### Pore sites: C state

**Figure 7:**
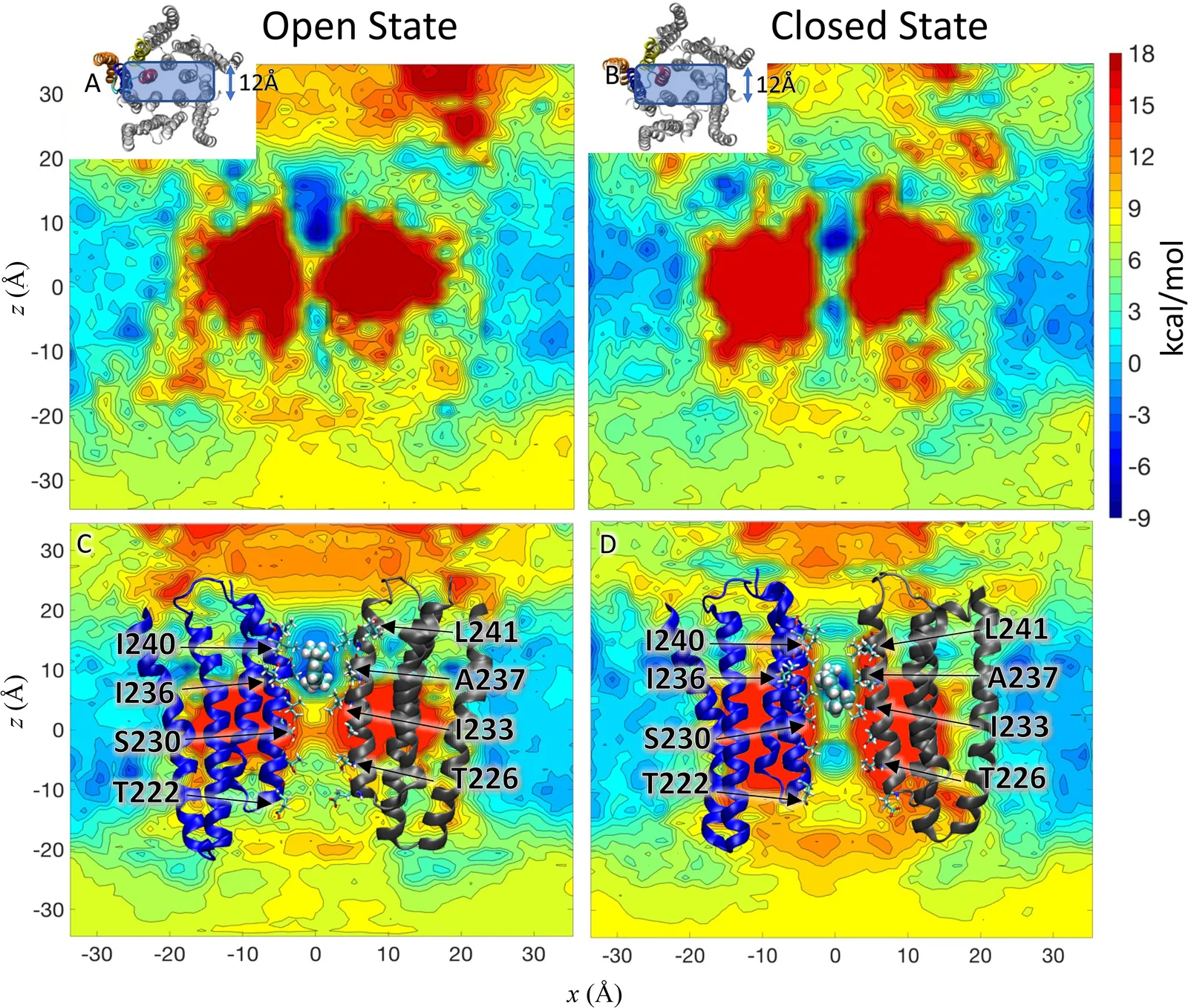
Change in the pore free energy surface between O and C states. (A and B) *xz* projections of unsymmetrized O- (A) and C-state (B) free energy over the pore region; (C and D) *xz* projections of symmetrized O- (C) and C-state (D) free energy, with GLIC structure overlaid. Relevant pore residues are labeled.

As may be anticipated, the pore exhibits stark changes between the C and O states for propofol binding (Figure 4F versus 4C), which has been highlighted in Figure 7. The upper-pore minimum is of similar free energy (−6 ± 1 kcal/mol) to the O state but is more localized. However, the lower-pore minimum is now more pronounced than in the O state, with a similar free energy (−2 ± 1 kcal/mol), but shifted to a lower *z* value, owing to the collapse of the pore. In the pore, there are rings of identical residues that contact propofol, as visualized in Figure 5E, being similar to the O state. The key residues are I240, A237, I233, and S230 (Figure S6D). Overall, interaction energies in the Pore sites are very similar to those in the O state. H-bonds between propofol and the pore-lining residues were nonexistent (Table S2), owing to the hydrophobic nature of the pore. Water interactions were reduced in magnitude compared with the more hydrated O-state pore (Figure S6E), as expected, with reduced number of phenol-water H-bonds (17% ± 18%; Table S2).

### Multi-propofol cooperative effects in the inter-subunit sites

#### Multi-propofol MTD sampling and pathways

Previous studies of GLIC have suggested the potential to bind two anesthetic molecules in the GLIC pore simultaneously.^54^ The binding of multiple anesthetic molecules to the GLIC Inter site has also been observed in experiment,^38^ where co-crystallization of propofol with F238′A/N239′A GLIC resulted in the each of the subunits being occupied. Moreover, strong dependence of modulation for wild-type, as well as mutant GLIC (spanning weak-strong potentiation followed by inhibition^38^) on anesthetic concentration suggests that there could be cooperative effects with binding to the TMD.

**Figure 8:**
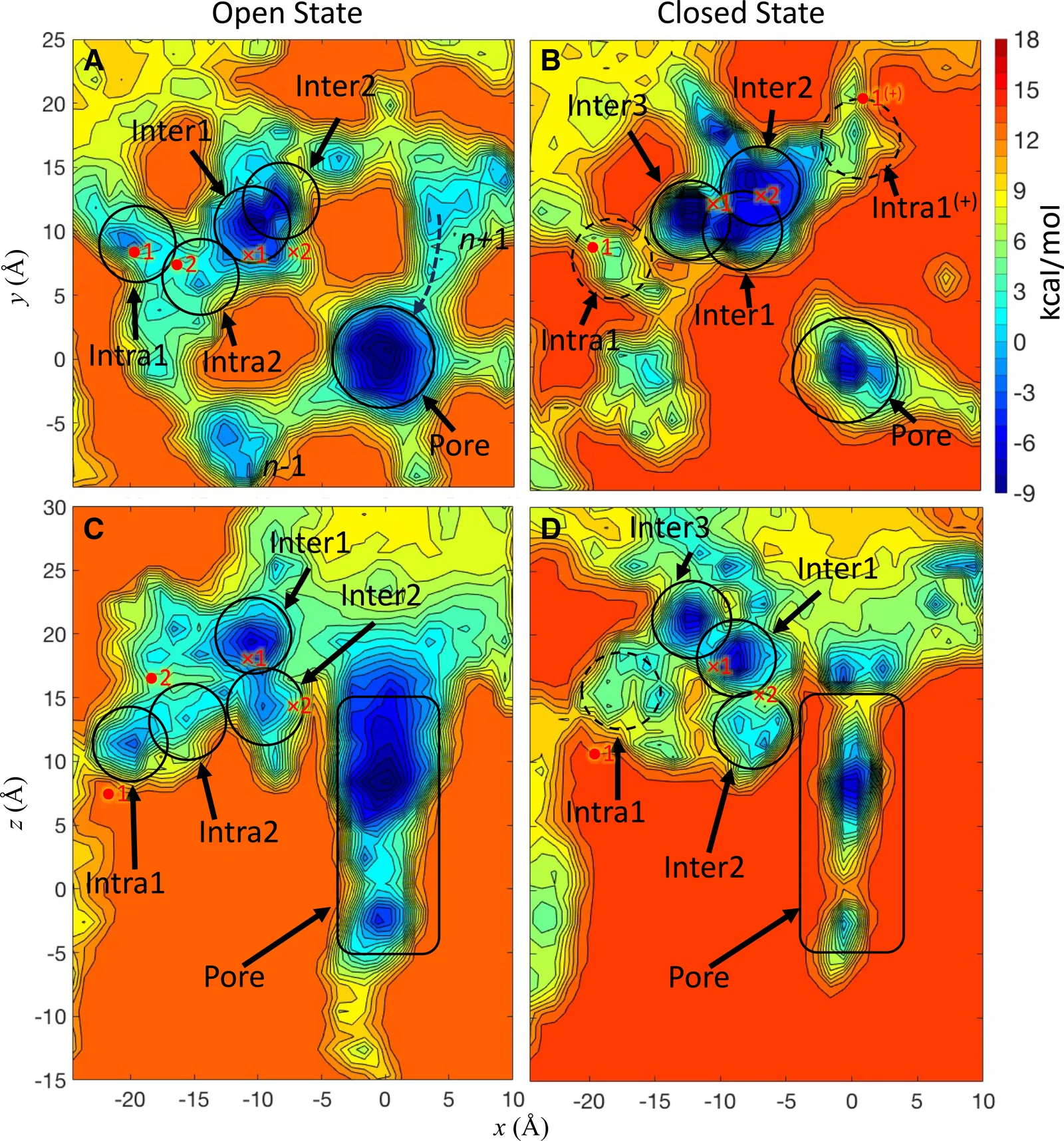
Multi-propofol free energy surfaces for cooperative effects involving the inter-subunit sites. (A and B) O- (A) and C-state (B) TMD subunit *xy* free energy projections. (C and D) O- (C) and C-state (D) *xz* projections. Errors are shown in Figures S4C and S4D. Site locations from the main MTD simulations (Figures 3 and 4) are indicated with red symbols ( for Intra and  for Inter), revealing shifts in the sites. Due to limited exploration of the membrane in these more focused multi-propofol MTD simulations, there exists an arbitrary additive constant, chosen by setting the deepest minimum to match Figure 4.

Given the results from the single-propofol MTD for propofol binding to the Inter site (seemingly more stable in the C compared with the O state; Figure 4D versus A), in contrast to the experimental suggestion of potentiation via this site,^36^ we explore here a limited study of cooperative effects involving the Inter sites. Moreover, the location of the Inter site, between adjacent subunits, may promote such inter-subunit cooperativity. Further studies would be needed to explore other cooperative effects. Additional simulations have been carried out where four of the five Inter sites were each occupied by a propofol molecule while binding to the fifth subunit was sampled via MTD. We simulated MTD within a restricted zone around that subunit, including only a small patch of surrounding membrane. For this reason, we do not set a reference 0 kcal/mol free energy in the membrane and instead focus on the positions and relative depths of the minima in Figure 8 (with additive constant adjusted to match the main sites with deepest minima in Figure 4). We will return below to compute free energies relative to the membrane using FEP.

Overall, the mutli-propofol free energy maps (Figure 8) suggest a similar set of sites but with differences to the single-propofol MTD results of Figure 4. The positions of the sites have clearly changed due to the presence of other bound propofol molecules. To highlight these changes, site locations from Figure 4 have been marked on Figure 8 as small red circles and crosses (Intra1 •1; Intra2 •2; Inter1 ×1; and Inter2 ×2). While the O state (Figure 8A) shows somewhat similar Intra sites and a similar Intra-Inter^(+)^ site pathway, there is a shift in the site locations for the Inter sites, now higher in the channel. We also observe that the O-state pore exhibits a broader region of low free energy than was seen in the single-propofol case of Figure 7A, suggesting the propofol molecules in all Inter sites have perturbed the M2 helices with which they remain in contact.

The region permitted to explore in these MTD simulations (extending beyond the one subunit into neighboring subunits) allows for examination of neighboring occupied Inter sites. For example, the negative *x*/negative *y* and positive *x*/positive *y* regions of Figure 8A extend into neighboring subunits, *n*−1 and *n*+1, respectively (as labeled). When the propofol molecule sampled these regions, a significant decrease in binding affinity (more positive binding free energy) compared with other sites was observed. For example, there is an absence of dark blue Inter sites in the neighboring subunits in Figure 8A. This implies that the occupied neighboring Inter sites cannot easily bind a second propofol simultaneously, despite possessing two separate minima (Inter1 and Inter2). An interesting outcome of this is the impact on the pathway into the Pore site. In the previous MTD with a single propofol in the O state (Figures 4A–4C), the pathway involved membrane-Intra1-Intra1-Inter1^(+)^-Pore with barriers as high as around 6 kcal/mol (Figure 4C). In the presence of propofol in the subunit, however, the propofol can sneak in without binding too strongly, as seen in Figure 8A (e.g., see dashed arrow near the *n*+1 subunit).

As seen in the single-propofol MTD sampling of Figures 4B and 4D, the Intra2 site is lost, and the Intra-Inter^(+)^ site pathway collapses in the C state (Figure 8B). Again, the lower free energy pathway relies on entrance into Intra1^(+)^ before moving to the Inter2 site (upper right region of Figure 8B). One notable change in the C state in Figure 8B (compared with Figure 4D and 4E) is the appearance of a new inter-subunit minimum, labeled “Inter3” (Figures 8B and 8D). The site is higher in the TMD, above a point approximately midway between Intra and Inter binding sites, and is located between residues R192 on M2^(−)^ and T249 and P250 on the (+) side of the M1-M2 loop. The absence of this Inter3 site in previous simulations indicates that it is likely the result of the neighboring subunits being occupied, leading to shifts in M2 helices. That is, neighboring propofol molecules bind to sites involving M2 and M1 helices on neighboring subunits, impacting the M2 and M1^(−)^ helices that form the Inter sites. The addition of the new Inter3 site leads to a broader low free energy inter-subunit region in the C state (Figure 8B).

#### Multi-propofol free energy perturbation and site analysis

The above exploration of the propofol binding surface relied on long MTD simulation times to attempt sample an equilibrium between the sites within GLIC’s TMD. For the multi-propofol MTD, the region sampled was restricted around the GLIC subunit, not extending far into the membrane, with the membrane-site equilibria remaining difficult to attain. An alternative approach is to sample at equilibrium along an alchemical coordinate that connects propofol in the membrane and in an individual site. Using knowledge of the locations of the sites from the multi-propofol MTD, quantitative calculations of free energy difference (relative to the distant membrane; see materials and methods) have been performed. Here, we report FEP calculations for each TMD site (Intra1, Intra2, Inter1, Inter2, Inter3, and Pore) within O or C GLIC states of the multi-propofol system, along with analysis of the residues contributing to the formation of each site.

Differences in free energies and residue interaction energies are expected between the previous single-propofol MTD and the multi-propofol FEP, primarily because of the cooperative effects of propofol molecules. We acknowledge, however, that differences may also arise from the different sampling approaches. In contrast to the MTD that relied on equilibrium sampling across the entire protein, requiring long simulation times (∼40 × 0.8 μs) to capture free energies across all sites (Figure S3), FEP used a dedicated set of simulations that independently focused all of their sampling (up to 20 × 0.2 μs; Figures S7 and S8) within each site. Results from multi-propofol FEP and single-propofol MTD are compared below with this in mind.

#### Intra-subunit sites: Multi-propofol, O state

The Intra1 free energy relative to bulk membrane was calculated to have a ΔG of –0.3 ± 0.5 kcal/mol (see Figure S7 for convergence), while the Intra2 ΔG was a deep –7.0 ± 0.5 kcal/mol. The FEP simulations thus reveal a much more attractive Intra2 site. The fact that the Intra2 free energy is more attractive likely arises from the depth of this site into the protein where interactions rely almost entirely on M1 helix residues that have been perturbed by adjacent propofol binding (in contrast to Intra1, which is more at the periphery of the TMD). The fact that the Intra1 site maintained a smaller free energy magnitude (relative to the membrane) may be expected because it is the first accessible site from the lipid bilayer and is expected to exhibit a similar free energy for a lipophilic molecule like propofol.

The key Intra-site residues can be identified from the interaction energy analysis in Figures S9A and S9B. For Intra1, these include I202, I258, T255, and M205. For Intra2, propofol interacted with Y197, V242, P120, I201, and T255, being largely the same as what was sampled in the main single-propofol MTD simulations. The water-propofol interaction energies (Figure S6E, right) in Intra1 and Intra2 are similar and exceed those observed in single-propofol MTD simulations (Figure S6E, left), suggesting perturbations of the TMD due to propofol molecules in adjacent subunits may draw in more water molecules into this region.

#### Inter-subunit sites: Multi-propofol, O state

The O-state Inter1 and Inter2 ΔG values are –1.5 ± 0.2 and −4.0 ± 0.4 kcal/mol, respectively (Figures S7C and S7D), both being attractive relative to the membrane, consistent with what was suggested by the multi-propofol-MTD results in Figure 8A. In the Inter1 site, key residues (Figure S9C) on the (−) side subunit include Y197^(−)^, I201^(−)^, and N245^(−)^, while on the (+) subunit, they include K248^(+)^ and E243^(+)^. For the Inter2 site, key residues on the (−) side (Figure S9D) were identified as N200^(−)^, P204^(−)^, and I201^(−)^, while on the (+) side, E243^(+)^, Y263^(+)^, and I259^(+)^ were important. These results reaffirm the strong interactions between propofol and N200, I201, and E243 from the main MTD simulations. The interaction energy with water in the Inter1 site in the O state (Figure S6E, right) is similar to that from MTD but is lower for the Inter2 site, which has moved, affecting its hydration.

#### Pore sites: Multi-propofol, O state

The FEP simulation of the O state pore did not attempt to sample the two disparate minima that flank the long channel pore. Instead, the propofol was trapped in the middle-upper pore. In this site the calculated ΔG was −9.3 ± 0.3 kcal/mol relative to the membrane, apparently consistent with the multi-propofol MTD of Figure 8C. Strong interactions were observed with A237, I233, I240, and S230 (Figure S9E), consistent with the single-propofol MTD analysis, albeit with stronger A237 interaction. The average water interaction energy is consistent with the single-propofol result (Figure S6E).

#### Intra-subunit sites: Multi-propofol, C state

In the C state, the Intra1 site is not as well defined in Figure 8B, with an elevated free energy (ΔG = +1.1 ± 0.3 kcal/mol) that is less well converged (Figure S8A, left) with a less-well-defined peak in its distribution (Figure S8A, right). Despite the collapse of the Intra2 minimum in the multi-propofol MTD, FEP was computed in this location, yielding a ΔG of −3.0 ± 0.5 kcal/mol, again not capturing a well-defined site (Figure S8B, right panel), but converging to a more negative free energy than that expected from Figure 8B. The C state Intra1 involved interactions with T255, I259, L246, and I202 (Figure S10A), being similar to what was seen in MTD, with the notable addition of L246. Intra2 included T255, I202, I258, M205, and Y254 (Figure S10B). The Intra1 site again experiences low hydration (Figure S6E, right), likely explaining the elevated free energy in this site in the C state.

#### Inter-subunit sites: Multi-propofol, C state

FEP simulations of the two C-state Inter1 and Inter2 sites in the multi-propofol-GLIC system revealed ΔG values of −3.9 ± 0.5 and −3.8 ± 0.3 kcal/mol, respectively (Figures S8C and S8D). Though the value for Inter1 is more negative than in the O state (−1.5 ± 0.2 kcal/mol), the value for the Inter2 site is more positive than the value in the O state (−4.0 ± 0.4 kcal/mol); though within errors. This suggests some shift favoring the O state for Inter2 compared with the single-propofol MTD, being more consistent with experimental suggestions of potentiation.

The major contributors to C-state Inter1 binding on the (−) subunit were I245^(−)^, N200^(−)^, and Y197^(−)^ (Figure S10C). On the (+) subunit, the key residues were K248^(+)^, L246^(+)^, and E243^(+)^. The Inter2 (−) contribution was dominated by N200^(−)^, I201^(−)^, and N245^(−)^ (Figure S10D). The (+) subunit interactions of Inter2 were dominated by L246^(+)^, I259^(+)^, and E243^(+)^. These residues are similar to those seen in the single-propofol MTD, with differences especially coming from the (+) subunit, including F260^(+)^, M252^(+)^, and G256^(+)^ for Inter1 and F260^(+)^ and G256^(+)^ for Inter2 (Figures S6B and S6C). These differences can be explained by the shifted Inter minima in Figure 8B. The C-state Inter1 and Inter2 sites had similar hydration energies, both with stronger hydration than the O state (Figure S6E).

The new inter-subunit minimum in the C state, named Inter3, has an elevated free energy +1.2 ± 0.2 kcal/mol relative to the membrane, being less attractive than was suggested by the multi-propofol MTD in Figure S8E. The residues that make up this site on the (−) subunit include R192^(−)^, N245^(−)^, and Y197^(−)^, while on the (+) subunit, they include P250^(+)^, T249^(+)^, and D32^(+)^ (Figure S10E). Two residues, R192 and D32, are particularly interesting, because of their state dependence and vital role in channel gating, where they form a salt bridge in the O state that is broken in the C state.^22^^,^^24^^,^^81^ Some of the residues involved in this new Inter3 pocket, such as N200 and E243, were also associated with binding in the main Inter sites. The Inter3 minimum is found high in the TMD (Figure 8B) and is able to access residues at the bottom of the ECD (including D32). As a result of the polar and charged nature of the site, it led to a strong propofol hydration energy (Figure S6E, right).

#### Pore sites: Multi-propofol, C state

The C state Pore site ΔG was −8.3 ± 0.4 kcal/mol (Figure S8F), again being the deepest free energy minimum in the channel. Key interactions include I240, A237, and I233 (Figure S10F), consistent with those seen in the upper pore in the single-propofol MTD. The propofol-water interaction energy was small (Figure S6E, right), as seen in the single-propofol MTD simulations and expected for a dewetted C-state pore.

## Discussion

In this study, we investigated the binding of propofol to the prototypical pLGIC protein GLIC, describing its interactions and modulatory effects to help understand the mechanisms of protein-mediated general anesthesia. Unbiased sampling was unsuccessful in mapping out the propofol binding landscape, though it did give some information on the partitioning into the membrane and the protein’s TMD interface, as well as binding to the ECD, including a previously suggested ketamine binding site.^63^^,^^65^ Enhanced sampling simulations were, however, required to explore the binding locations within the TMD, revealing the interactions and free energies of binding to each site. Additional enhanced-sampling simulations also examined binding in the presence of propofol molecules in neighboring subunits, revealing cooperative effects communicated via perturbations to TMD structure. Together, we now piece together what were the main findings in terms of the sites found and their likely contributions to channel modulation.

### Propofol binding sites are state dependent

MTD simulations were able to explore the entire pentamer of GLIC and each of its TMD-based sites. The three main sites were in the intra-subunit (Intra), inter-subunit (Inter), and pore (Pore) regions. Each of these main sites was bifurcated into two adjacent sites: Intra1 and Intra2; Inter1 and Inter2; and upper and lower Pore. Table 1 summarizes the free energies and important interactions in the different sites, with key residue interactions discussed in the supplemental information.

The strongest binding was seen to occur within the Pore, and particularly in the upper pore region, with a deeper (and broader) free energy minimum there. This is in contrast to previous suggestions that the Pore site for propofol is located near the center of the TMD,^33^^,^^45^^,^^53^^,^^54^ similar to that seen for bromo-lidocaine,^67^ but is consistent with the presence of peripheral density peaks near the upper and lower pore seen previously for propofol and isoflurane.^53^ Channel closure did not affect the depth of the free energy minimum within the upper pore site significantly, but it did lead to its displacement (by over 5 Å) from the periphery to deeper into the pore, likely further occluding the narrowed hydrophobic pore in the C state. Channel closure also led to a more pronounced and deeper free energy minimum at the lower end of the pore. Examination of the pore region within the O and C states showed influence from I240, A237, and I233, as well as S230, seen in the X-ray structure.^33^ There was increased prominence of T222 and T226 residues in the lower C-state Pore. This state dependence differs from that seen previously where peaks in the lower and upper pore primarily arose from multiple propofol binding, and where binding in the upper pore was preferred in the C state.^53^ This difference is possibly a result of the fairly short (50–100 ns) unbiased simulations used in that previous study.^53^ Similarly, the existence of a density peak in the middle of the channel may have arisen in that study from the initial placement near the experimentally determined site. The lower-pore residues T222 and T226 could be subject to experimental mutagenesis examination for propofol modulation of GLIC.

Binding to the other sites was strongly state dependent. Comparing the O and C states (summarized with blue and red entries in Table 1), we see that channel closure, with corresponding movements of M2 helices toward the channel axis, led to less favorable binding within the intra-subunit sites, even eliminating the Intra2 site. Occlusion of the Intra site in the C state is consistent with experiments that suggest lack of anesthetic access to that pocket in the resting state.^50^ The Intra1 and Intra2 minima showed strong interactions with key residues (T255 on the M3 helix, M205 on M1, and V242 on M2) that have previously been identified in experiments,^14^^,^^38^ primarily identifying Intra2 as the experimentally defined intra-subunit binding site. However, the contributions of each of the residues varied between states. In the O state, T255 interacted strongly in both Intra1 and Intra2, while V242 contributed only to Intra1 and M205 only to Intra2. M205 plays a central role in the experimentally defined Intra site and has been mutated to stabilize propofol in that site to enhance potentiation.^38^ In the C state, the Intra2 site was eliminated, leaving Intra1 interacting with T255 (M3) and M205 (M1) but not with V242 (M2). This can be explained by the movement of the M2 helix into the pore away from the adjacent M1^(−)^ during channel closure (Figure 1C). Residues I202 and I258 also exhibited stronger interactions with propofol in both states than the experimentally identified residues. The strength of I202 (M1) and I258 (M3) interactions was even greater in the C state, implying that M2 movement has freed up these residues to interact with propofol. The importance of M1 residue I202 in the Intra site has been noted previously,^14^ leaving the M3 I258 residue as a possible new test for experimental mutagenesis.

Channel closure appeared to lead to increased affinity for inter-subunit sites for the MTD simulations of single-propofol binding. But this was not the case in the multi-propofol MTD simulations, suggesting a possible correlation effect for inter-subunit propofol modulation (see allosteric modulation section below). Regarding key interactions, in the O state, N200 (M1) and E243^(+)^ (M2) interacted most strongly with propofol, consistent with experimental findings,^38^ with both the Inter1 and Inter2 sites sharing residues in common with those inferred from experiment. In the C state, I259 and F260 on M3 appear to dominate. A238 and A239 residues are only weakly interacting owing to the F238A/N239A double mutation (eliminating aromatic and polar groups) used to open up the Inter site. Instead, much more interaction was seen from N245 in the Inter1 minimum and L241 in Inter2. These residues along with N200 and E243^(+)^ appear to be the primary contributors to the Inter binding site. Also notable is the involvement of K248^(+)^ on the M2-M3 loop in the O state, which changes to M252^(+)^ (M2-M3 loop) interaction in the C state, which is likely due to the movement of the M2-M3 loop during gating seen experimentally.^24^ There are therefore several residues that are subject to experimental verification involving Inter site binding.

### Cooperative effects of multiple propofol binding

Experimentally, modulation varies dramatically depending on the concentration of propofol.^38^ This may suggest a possible role for multiple binding of propofol molecules, as in seen in high-resolution structures (e.g., Fourati et al.^38^). As an illustration, we focused on the Inter sites, given that they interact directly with neighboring subunits and may be most likely to show correlation between adjacent binding events. Additional MTD simulations were undertaken that examined the effect of multiple propofol molecules simultaneously bound to the Inter sites between all subunits within the O and C states of GLIC. The existence of neighboring propofol molecules was seen to influence the free energies and the positions of inter-subunit sites. A novel Inter3 binding pocket emerged in the C state, positioned high in the TMD between Intra and Inter^(+)^ sites. This site featured interactions with both R192 and D32 residues, which are known to form a salt bridge in the O state and not the C state.^24^ With the salt bridge broken in the C state, the lower β sandwich in the ECD expands, which causes the M1 helix to form a kink and break a pair of stabilizing salt bridges between S196-E243 and N200-N239, influencing inter-subunit structure. Interestingly, binding of halothane at the ECD-TMD interface has previously been seen to interrupt this same R192-D32 salt bridge.^68^^,^^93^ This salt bridge could be the subject of future testing for anesthetic sensitivity, but may be complicated because of its critical role in gating.

### Pathways connecting the binding sites change with conformational state

By exploring the GLIC TMD, the simulations identified connecting pathways between sites that changed between conformational states. In the O state, propofol entered the Intra site from the membrane, initially occupying the Intra1 minimum. The observation of a membrane-embedded tunnel into the TMD is consistent with the Meyer-Overton hypothesis (anesthetic potency dependency on lipophilicity) as has been observed in previous simulations (of desflurane^62^). Once inside the TMD Intra1 site, propofol ascended toward the Intra2 minimum higher in the TMD. From there, the molecule could access the path to the Inter^(+)^ region and transit clockwise to Inter1. At this point, propofol could either proceed to the Inter2 minimum or penetrate into the Pore site. While previous studies have predicted an Intra-Inter^(+)^ linkage, the transition from Inter to Pore has been uncovered by these simulations.

The pathways connecting the sites in the C state contain several notable differences to the O state. The M2 shift that occurs in the C state obstructs the previous Intra-Inter^(+)^ pathway and causes the Intra2 minimum to collapse. Instead, propofol gains access to the pore through the Intra1 minimum and instead travels toward Inter2^(−)^ (on the preceding subunit). While M2 inward motion leads to closure of the previous pathway, it also reduces the Intra-Inter^(−)^ barrier, facilitating this new transition. Additionally, the movement lowers the free energy barrier that separates the Inter site from the surrounding lipid membrane. Consequently, propofol was observed entering and exiting the channel from both Intra1 and Inter1 sites.

Multi-propofol simulations revealed a shortcut into the pore in the O state, where a propofol was observed to diffuse from the membrane into the region already occupied by propofol (which reduces the strength of binding of a second molecule) and straight into the pore. In contrast, this pathway was eliminated within the C state. The occurrence of different pathways of anesthetic movements due to the binding of multiple molecules indicates the significant role concentration may have in shaping the binding landscape, and it reveals new pathways that may be targets for novel anesthetics. Moreover, it suggests an increased rate of pore binding at higher concentration, with cooperativity between multiple propofol molecules, that could be examined with experimental kinetics.

### Induced-fit modulation of the pore

Propofol binding alters the local shape of the GLIC protein when it binds, with the potential to affect function. An obvious candidate is the effect of binding on the pore size to control ion conduction. A good indicator of whether GLIC is in an O or C state is the distance separating the M2 and M1^(−)^ helices^13^ (Figure S11). While histograms showed that simulations that started in the O state remained open and simulations that started in the C state remained closed, regardless of propofol binding, there were small changes, depending on which site was occupied. With no propofol bound in the O state (Figure S11A), the pore was open, with a small component with lower M2-M1^(−)^ distances that are considered “wide open” (distance < ∼11.5 Å), not previously sampled in solutions for the pathways of gating^22^ and likely arising from the extended MTD simulations performed. Binding in the Intra sites led to a shift away from lower distances to the main “open” peak in the distribution (between ∼11.5 and 15Å), with similar, but lesser effects when binding to the Inter sites or the pore. In all sites, binding caused a splitting of the main M2-M1^(−)^ distance peak into two peaks, including a minor component for a less open state (distance ∼14.5Å). This localized peak represents the channel attempting to gate (transition to the C state), but being prevented from doing so due to the imposed flat-bottom restraint that maintains the O state conformation (see materials and methods; the helix was forced back if it passed a distance of 14.5 Å). Thus, there exists a local adjustment of structure that tends to close the pore with propofol bound, to some minor degree, but where the major population corresponds to a maintained open pore.

In the Intra site, propofol interacts strongly with M1^(−)^ helices (see Figure 1D; Table 1), likely “pulling” it outward to drive larger M2-M1^(−)^ distances, weakening M2 interactions with M1^(−)^. In the case of the Inter sites, the presence of propofol between two subunits means that it resides close to the M2-M1^(−)^ pair, interacting strongly with both helices (see Figure 1D; Table 1). M2-M1^(−)^ showed a small shift toward higher distances, likely a result of the resident propofol wedging between the helices. Without a ligand binding to an Inter site, experimental studies^38^ indicate that N200^(−)^ (M1) and E243^(+)^ (M2) form a bridge mediated by a water molecule. Table 1 shows these residues now interact strongly with propofol, likely weakening the M2 to M1^(−)^ interaction. A closer look at the Inter sites in terms of what binding does to the same subunit and a distant subunit (Figure S11A; upper insets) shows a discrepancy, where the same subunit (*n*) is pushed toward higher M2-M1^(−)^ values, whereas the opposing subunit (*n*+2) remains firmly open.

In the C state simulations (Figure S11B), the pore remained consistently closed, regardless of propofol binding, albeit with some elongation of the tail of the distribution to more-closed states when propofol was bound to the Pore site, a result of the mostly hydrophobic ligand tending to attract the hydrophobic M2 helices (notably interacting with I240, A238, and I233, as well as T222 and T226 in the lower pore) that line the pore.

This analysis suggests that local adjustments in protein structure due to propofol binding occur and that they generally tend to shift the distribution of M2-M1^(−)^ distances. However, the major component within the O state is that corresponding to an open pore. This is consistent with previous simulations that used unbiased simulations to observe some shift to a closed pore upon asymmetric binding of desflurane to GLIC.^62^ However, in our simulations, which are trapped in the O state, as done in previous simulations to maintain one conformational state (e.g., LeBard et al.^53^), no evidence of strong shift toward a closed-pore state was observed.

### Allosteric modulation

Propofol may modulate the ion channel by binding more favorably in one conformational state than in another. If it binds more favorably to the conducting state, then it should potentiate the channel (if it does not block function in some way, such as occluding the ion-conducting pore), whereas if it binds more favorably to nonconducting states, it will be inhibitory. Experimentally, modulation is measured based on, e.g., change in EC_10_ current (which occurs around pH 5.5 for wild-type GLIC) with a set concentration of anesthetic (e.g., 30 μM propofol).^38^ This pH more closely aligns with the pH 4.6 condition that promotes the O state than with the neutral pH used experimentally to promote the C state. While pH 5.5 may be considered mid-range, it is not necessarily applicable to both pH values, simply because, while the pH_50_ (the pH at which half maximal current is achieved) of GLIC is 5.1,^94^ the activity of the channel is sigmoidal, transitioning from nearly fully active at pH 4.6 to fully inactive at pH 7.^94^ It is therefore likely that a calculated favorable free energy of binding in one state/pH over the other state/pH may not correspond to experimental modulation at mid-range pH. Moreover, modulation is seen experimentally to be highly sensitive to propofol concentration. Wild-type GLIC inhibition grows steadily as the concentration of propofol increases from 1 μM to 300 μM.^38^ One cannot therefore expect exact quantitative agreement with experiments for single-molecule binding simulations. The simulations should, however, shed light on how such modulation may occur.

It is understood that propofol inhibits GLIC,^38^^,^^52^ but it does so by binding to the Pore of the channel, with the other TMD sites being potentiating in nature.^38^ Analysis of Table 1 for the free energies within the double-mutant F14′A + N15′A GLIC channel suggests that the Intra sites are potentiating. That is, via a thermodynamic cycle, the free energy difference between C and O conformational states in the presence and absence of propofol equals the difference in the binding free energies of propofol to C and O states. In this Intra-site case, there is preferential binding to the O state, and therefore, propofol binding preferentially stabilizes that state. Note that while this shift between O and C states exists for both Intra1 and Intra2 sites, the free energy is considerably lower in the Intra1 site, and this site therefore dominates, as is seen in the Boltzmann-averaged summary of Table 2 that combines Intra1 and Intra2 into one Intra binding location with a −3 ± 3 kcal/mol net change. The effect grows to −8.1 ± 0.6 kcal/mol in the multi-propofol calculations, signifying a strong potentiating effect.

**Table 2:** Combined free energies for binding to Intra, Inter, and Pore regions

	Δ*G (kcal/mol)*			$\Delta \Delta G_{C\to O}$*(kcal/mol)*
	O	–	C	
	Single-propofol MTD (multi-propofol FEP)			
Intra	−4 ± 2 (−7.0 ± 0.5)	• (←)	+0 ± 2 (+1.1 ± 0.3)	−3 ± 3 (−8.1 ± 0.6)
Inter	−1 ± 2 (−4.0 ± 0.4)	• (•)	−4 ± 2 (−4.3 ± 0.4)	+3 ± 3 (0.3 ± 0.6)
Pore	−6.7 ± 0.7 (−9.3 ± 0.3)	• (←)	−6 ± 1 (−8.3 ± 0.4)	−1 ± 1 (−1.0 ± 0.5)

For each site, the binding free energy (relative to the membrane) from single- (MTD) and multi-propofol (FEP; bracketed) simulations is specified, based on an equilibrium combination of 2 or 3 subsites from Table 1 at pH 4.6: i.e., Intra1 and Intra2; Inter1, Inter2 and possibly Inter3; as well as Pore(mid-upper) and Pore(lower). Subsites were combined via $\Delta G=-k_{B}T\;\ln\left(e^{-\Delta G_{1}/k_{B}T}+e^{-\Delta G_{2}/k_{B}T}+e^{-\Delta G_{3}/k_{B}T}\right)$, along with error, $\delta \Delta G=\left(\frac{e^{-\Delta G_{1}/k_{B}T}\delta \Delta G_{1}+e^{-\Delta G_{2}/k_{B}T}\delta \Delta G_{2}+e^{-\Delta G_{3}/k_{B}T}\delta \Delta G_{3}}{e^{-\Delta G_{1}/k_{B}T}+e^{-\Delta G_{2}/k_{B}T}+e^{-\Delta G_{3}/k_{B}T}}\right)$. Errors in $\Delta \Delta G_{C\to O}$ come from the square root of the summation of variances. n.b. Errors from MTD results arise from symmetrization around the pentamer and are inherently larger than statistical errors from FEP with propofol trapped in a site. Arrows indicate an apparent direction of change based on the minimal free energies (“•” meaning no change within error).

Experimentally, the Intra site is considered to be potentiating, according to several lines of evidence. Propofol was only observed bound to GLIC within the O state in X-ray crystallography, with the Intra site being remodeled within the C (and locally closed LC) states.^24^^,^^49^ That is, the Intra site in the C states is presumably less able to bind the anesthetic, as has also been implied from thiosulfonate-labeling studies.^50^ Experiments have examined the response of residue M205, which is seen to be important in forming the site (Table 1), by studying the effects of GLIC mutant M205W on the actions of anesthetics propofol and bromoform.^38^ This mutation had the effect of making the channel more readily potentiated by propofol or bromoform. As such, the Intra site has been determined to be potentiating, consistent with our calculations.

Experimentally, the Inter site has also been suggested to be potentiating,^36^ based on the apparent stabilization of the O state GLIC F14′A crystal structure in the presence of ethanol and bromoform. It has been suggested that propofol bridges more contacts between subunits in the O state,^38^ consistent with our analysis (Table 1). Yet, the free energy maps for single-propofol binding to the O and C states of the double mutant F14′A + N15′A GLIC channel in Figures 4 and 8 appear to tell a different story, with apparently deeper free energy minima in the inter-subunit region in the C state. However, one must consider the errors (especially in the MTD results influenced by dynamic subunit asymmetry) and the preferential binding to Inter1 over Inter2 in the O state (such that Inter1 dominates in the Boltzmann average). The summary data in Table 2 suggests that the free energy difference is +3 ± 3 kcal/mol, being positive but within errors. In MTD and FEP calculations of propofol binding to Inter sites in the presence of 4 other propofol molecules in neighboring inter-subunit sites, we observed major shifts in the free energies of binding and, in particular, more favorable binding in the Inter sites in the O state than was seen in single-propofol MTD. In the multi-propofol FEP results the free energy difference is now just +0.3 ± 0.6 kcal/mol. This suggests that there is no clear potentiating or inhibitory result for the Inter sites and that it likely depends on the cooperation of propofol across subunits. Given the experimental observation of co-crystallization of propofol with the double mutant F14′A + N15′A GLIC channel at all five subunit interfaces within the O state,^38^ we tend to favor a conclusion that the thermodynamic modulation of the channel from the Inter site is marginal, but it may still be potentiating, in-line with inferred effects from structure-based studies.^36^^,^^38^

The Pore has been determined experimentally to be a conserved site for pLGIC inhibition^38^ for propofol and other anesthetics.^39^^,^^95^^,^^96^ Our calculations (Table 1) suggest binding of propofol with similar affinity to O and C states in the upper pore, consistent with past estimates using FEP(54). However, binding shifted toward the center of the hydrophobic pore (minimum displaced by over 5 Å) in the C state. This is consistent with past studies suggesting movement of isoflurane into the closed pore, despite its dewetting.^66^ Previous simulations^53^ and experiments^97^ have suggested preferred binding of dimers of anesthetic molecules (isoflurane or propofol) inside the pore. This led to stronger pore-binding affinity that has been suggested to be partially offset by binding to potentiating sites in a multimodal mechanism.^53^ Further MTD simulations of multiple propofol binding thermodynamics within the pore may be needed, as well as simulations to demonstrate block of ion conductivity in the presence of propofol monomers and dimers. However, while propofol has similar affinity for both O and C states inside the pore, occlusion of the open ion-conducting pore seems a logical mechanism of action.

It has been previously suggested that the Pore may represent a lower affinity site than other sites in the TMD.^38^ This was used as a means for explaining the growing potentiation with propofol concentration for the M205W mutant GLIC channel, which turns inhibitory as the Intra sites saturate, and the Pore site begins to fill with propofol.^38^ In contrast, the pore minimum was seen here to be deeper and broader, appearing as the single highest affinity site (Table 1; Figure 4). An alternative explanation for the functional data comes from the fact that there exist five Intra sites versus just one Pore site, and that affinities of the Intra and Inter sites change upon binding of anesthetic molecules to these multiple sites. Binding of a single propofol to the pore may occur first without blocking it, but successive binding of molecules to the pore may not be favored at low concentration (multi-propofol binding to the pore not calculated here). Instead, Intra (and Inter) sites may be filled successively, lowering the free energy of the O state (with the preference over the C state increasing in the case of multi-propofol occupation; Table 2) until those sites are saturated, after which propofol molecules may continue to accumulate within the Pore region, fully occluding the ion-conducting pathway and inhibiting function.

### CONCLUSION

After more than a century of research into how anesthetics work,^98^ structural resolution of pLGICs, combined with enhanced-sampling simulation methods, can provide a route toward understanding protein-mediated general anesthesia and for developing improved anesthetic compounds. The simulations carried out here have aimed to complement structural and functional experiments by providing a molecular-level basis for understanding general anesthetic modulation of an ion channel. These simulations have revealed previously undescribed interactions and pathways within the prototypical channel GLIC that may be tested with new experimental mutagenesis and anesthetic sensitivity studies. Calculated free energy changes are consistent with bimodal modulation via competing sites that act to inhibit via the pore or potentiate via intra-subunit sites. Binding to inter-subunit sites revealed no clear evidence for allosteric modulation, but was seen to be dependent on cooperative binding to neighboring subunits, which may help understand the concentration dependence of general anesthetic modulation. The findings help explain modulation of the Cys loop superfamily of channels and may enable new studies that can target pLGICs with novel anesthetics.

## Data availability

Simulation data (US, flooding, multi-walker metadynamics, and FEP) for each system/protein conformational state can be made available upon request to the corresponding author. Each set of simulations consists of extensive highly replicated enhanced-sampling data that may require special methods for unbiasing to allow for trajectory analysis.

## Acknowledgments

We acknowledge discussions with Brett Cromer and are grateful for support from the 10.13039/501100000923Australian Research Council (DP210102405 and DP2201035501), 10.13039/501100000925National Health and Medical Research Council (APP1104259 and APP1141974), National Computational Initiative (dd7), and from the Medical Advances Without Animals Trust for their scholarship support for A.J.D.

## Author contributions

A.J.D. performed research, analyzed data, and wrote the paper; B.L. performed research; T.W.A. designed research, analyzed data, and wrote the paper.

## Declaration of interests

The authors declare no competing interests.

## Supplemental information

Document S1. Figures S1–S11 and Tables S1 and S2

Open state metadynamics convergence corresponding to Figure S3a

Document S2. Article plus supplemental information
