# Supplementary material for: General anesthetic modulation of a pentameric ligand-gated ion channel explored with simulations
Source: Biophys J. 2026 May 15;125(16):4462–86. doi: 10.1016/j.bpj.2026.05.007 (PMC13507486; doi:10.1016/j.bpj.2026.05.007)
Supplement: Supplementary file 1 [file mmc1.pdf]

Biophysical Journal, Volume *125*

Supplemental information

General anesthetic modulation of a pentameric ligand-gated ion channel explored with simulations

Adam J. Dymke, Bogdan Lev, and Toby W. Allen

Supplemental Information for: ***General anaesthetic modulation of a pentameric ligand-gated ion channel explored with simulations***

Adam J. Dymke[1], Bogdan Lev[1] and Toby W Allen[1]

[1] School of Science, RMIT University, Australia.

Propofol-residue interaction energies in different sites

Here we summarise the key interaction energies that are discussed in the Results section. The interaction energies between propofol and all protein residues for each transmembrane domain (TMD) site are graphed in Figs.S5 and S6 (Open (O) and Closed (C) states, respectively, from single propofol Metadynamics (MTD)) and in Figs.S9 and S10 (O and C states, respectively, from multi-propofol Free Energy Perturbation (FEP)). The summary Table 1 (Discussion section) compares key residues involved for each conformational state of the channel.

<u>Single-propofol Metadynamics</u>

Intra-subunit sites:

O state *(Fig.S5A-B,S6E; visualised in Fig.5A-B):* Propofol in the Intra1 site interacted strongly with I202 (interaction energy –3.0 ± 0.3 kcal/mol), I258 (–2.0 ± 0.2 kcal/mol), I206 (–2.0 ± 0.1 kcal/mol) and water (–2.7 ± 0.6 kcal/mol). The Intra2 site was dominated by T255 (–2.9 ± 0.2 kcal/mol), Y197 (–2.5 ± 0.1 kcal/mol), I202 (–2.2 ± 0.4 kcal/mol) and water (–1.0 ± 0.5 kcal/mol).

C state *(Fig.S6A,S6E; visualised in Fig.6A):* Key Intra1 site interactions in the C state include I202 (–2.7 ± 0.2 kcal/mol), I258 (–2.2 ± 0.2 kcal/mol), T255 (–1.8 ± 0.2 kcal/mol), M205 (–1.5 ± 0.2 kcal/mol) and water (–1.7 ± 0.5 kcal/mol). The Intra2 site was non-existent in the C state.

Inter-subunit sites:

O state *(Fig.S5C-D,S6E; visualised in Fig.5C-D)*: Propofol in the Inter1 site was strongly bound to N245$^{(-)}$ (–2.6 ± 0.2 kcal/mol), Y197$^{(-)}$ (–2.5 ± 0.3 kcal/mol), I201$^{(-)}$ (–1.8 ± 0.2 kcal/mol) and water (–10.0 ± 0.9 kcal/mol). The (+) side interactions were dominated by E243$^{(+)}$ (–1.7 ± 0.2 kcal/mol) and K248$^{(+)}$ (–1.2 ± 0.3 kcal/mol). Inter2 shared key residues with Inter1 and was defined by N200$^{(-)}$ (–3.1 ± 0.3 kcal/mol), L241$^{(-)}$ (–2.4 ± 0.2 kcal/mol), I201$^{(-)}$ (–2.3 ± 0.3 kcal/mol) and water (–8 ± 1 kcal/mol). The opposing side featured E243$^{(+)}$ (–3.7 ± 0.2 kcal/mol) and I240$^{(+)}$ (–1.5 ± 0.2 kcal/mol).

C state *(Fig.S6B-C,S6E; visualised in Fig.6B-C):* Inter1 site propofol interactions involve N200$^{(-)}$ (– 2.6 ± 0.2 kcal/mol), L241$^{(-)}$ (–1.2 ± 0.1 kcal/mol) and N245$^{(-)}$ (–1.2 ± 0.2 kcal/mol) on the (-) side subunit. The key residues identified on the (+) subunit were F260$^{(+)}$ (–1.5 ± 0.1 kcal/mol), M252$^{(+)}$ (–1.3 ± 0.1 kcal/mol) and G256$^{(+)}$ (–1.3 ± 0.2 kcal/mol). Water interaction energy was –9 ± 2 kcal/mol. The Inter2 site is made up of N200$^{(-)}$ (–2.0 ± 0.2 kcal/mol), L241$^{(-)}$ (–1.5 ± 0.1 kcal/mol), P204$^{(-)}$ (–0.8 ± 0.1 kcal/mol) and N245$^{(-)}$ (–0.8 ± 0.1 kcal/mol), as well as I259$^{(+)}$ (–1.7 ± 0.1 kcal/mol), F260$^{(+)}$ (–1.6 ± 0.2 kcal/mol) and G256$^{(+)}$ (–1.4 ± 0.1 kcal/mol). The water interaction energy was –9 ± 1 kcal/mol in the Inter2 site.

Pore sites:

O state *(Fig.S5E,S6E; visualised in Fig.5E):* The key Pore site residues were I240 (–0.75 ± 0.05 kcal/mol), A237 (–0.7 ± 0.1 kcal/mol), I233 (– 0.4 ± 0.1 kcal/mol), S230 (–0.3 ± 0.1 kcal/mol) and water (–9.0 ± 0.8 kcal/mol).

C state *(Fig.S6D-E; visualised in Fig.6D):* Key residues are I240 (–1.0 ± 0.1 kcal/mol), A237 (–0.6 ± 0.1 kcal/mol), I233 (–0.6 ± 0.1 kcal/mol), S230 (–0.4 ± 0.1 kcal/mol) and water (–5.4 ± 0.8 kcal/mol).

Multi-propofol FEP

Intra-subunit sites:

O state *(Fig.S9A-B; S6E):* Key interactions for the Intra1 site include I202 (−4.1 ± 0.2 kcal/mol), I258 (−2.9 ± 0.1 kcal/mol), T255 (−2.1 ± 0.3 kcal/mol), M205 (−2.0 ± 0.05 kcal/mol) and water (−3.0 ± 0.6 kcal/mol). When inside Intra2, propofol interacted with Y197 (–3.5 ± 0.2 kcal/mol), V242 (–3.4 ± 0.2 kcal/mol), P120 (−2.7 ± 0.3 kcal/mol), I201 (–2.6 ± 0.1 kcal/mol), T255 (–1.8 ± 0.3 kcal/mol) and water (−3.7 ± 0.2 kcal/mol).

C state *(Fig.S10A-B; S6E):* Intra1 involved propofol interactions with T255 (−4.0 ± 0.2 kcal/mol), I259 (−2.5 ± 0.2 kcal/mol), L246 (−2.2 ± 0.1 kcal/mol), I202 (−2.1 ± 0.1 kcal/mol) and water (–0.8 ± 0.2 kcal/mol). Intra2 included T255 (−3.5 ± 0.3 kcal/mol), I202 (−3.2 ± 0.2 kcal/mol), I258 (−2.3 ± 0.1 kcal/mol), M205 (−1.4 ± 0.7 kcal/mol), Y254 (−1.2 ± 0.1 kcal/mol) and water (–2.9 ± 0.3 kcal/mol).

Inter-subunit sites:

O state *(Fig.S9C-D; S6E):* In the Inter1 site, key residues on the (–) side subunit include Y197$^{(-)}$ (–3.0 ± 0.2 kcal/mol), I201$^{(-)}$ (–2.3 ± 0.1 kcal/mol) and N245$^{(-)}$ (−1.8 ± 0.2 kcal/mol). On the (+) subunit, residues K248$^{(+)}$ (–5.0 ± 0.9 kcal/mol) and E243$^{(+)}$ (–1.9 ± 0.1 kcal/mol) contributed. Water contributed –7.5 ± 0.6 kcal/mol. For the Inter2 site, key residues on the (–) side subunit were N200$^{(-)}$ (–4.4 ± 0.3 kcal/mol), P204$^{(-)}$ (–2.7 ± 0.2 kcal/mol) and I201$^{(-)}$ (−1.8 ± 0.2 kcal/mol). On the (+) side of the Inter2 site, E243$^{(+)}$ (–3.7 ± 0.2 kcal/mol), Y263$^{(+)}$ (–2.2 ± 0.2 kcal/mol) and I259$^{(+)}$ (−2.0 ± 0.2 kcal/mol) were important, while water interaction energy was just −1.1 ± 0.1 kcal/mol.

C state *(Fig.S10C-E; S6E):* Inter1 binding on the (–) subunit included residues I245$^{(-)}$ (−3.6 ± 0.5 kcal/mol), N200$^{(-)}$ (−3.5 ± 0.1 kcal/mol) and Y197$^{(-)}$ (–2.1 ± 0.1 kcal/mol) (Fig.S10C). On the (+) subunit, K248$^{(+)}$ (–2 ± 1 kcal/mol), L246$^{(+)}$ (–1.3 ± 0.1 kcal/mol) and E243$^{(+)}$ (–1.1 ± 0.1 kcal/mol). The water interaction energy was –9.4 ± 0.5 kcal/mol. The Inter2 (–) contribution was dominated by N200$^{(-)}$ (–4.4 ± 0.3 kcal/mol), I201$^{(-)}$ (−2.3 ± 0.4 kcal/mol) and N245$^{(-)}$ (–2.2 ± 0.1 kcal/mol). The (+) subunit interactions were L246$^{(+)}$ (–1.3 ± 0.1 kcal/mol), I259$^{(+)}$ (–1.3 ± 0.2 kcal/mol) and E243$^{(+)}$ (–0.8 ± 0.1 kcal/mol). Water interaction was –8.0 ± 0.4 kcal/mol. The Inter3 site involved interactions on the (–) subunit with R192$^{(-)}$ (–4.7 ± 0.5 kcal/mol), N245$^{(-)}$ (−3.9 ± 0.3 kcal/mol) and Y197$^{(-)}$ (–3.1 ± 0.1 kcal/mol). Interactions on the (+) subunit include P250$^{(+)}$ (–2.1 ± 0.2 kcal/mol), T249$^{(+)}$ (–1.6 ± 0.1 kcal/mol) and D32$^{(+)}$ (–0.8 ± 0.1 kcal/mol), while water interaction was –6.4 ± 0.4 kcal/mol.

Pore site:

O state *(Fig.S9C-D; S6E):* Interactions included A237 (–1.0 ± 0.1 kcal/mol), I233 (–0.8 ± 0.1 kcal/mol), I240 (–0.6 ± 0.1 kcal/mol) and S230 (–0.2 ± 0.1 kcal/mol), with a strong interaction with water (–7.3 ± 0.6 kcal/mol).

C state *(Fig.S10F; S6E):* Interactions included I240 (–1.4 ± 0.1 kcal/mol), A237 (−1.3 ± 0.1 kcal/mol) and I233 (–0.7 ± 0.1 kcal/mol), with negligible water involvement (+0.20 ± 0.03 kcal/mol).

O state	Intra1	Intra2	Inter1	Inter2	Pore
Residues:	P120, Y197, I201, I202, M205, L206, V242, Y254, Y255, I258, I259, N307, A311	P120, S196, I198, V242, E243, N245, L246, Y255, I259	P120, G159, Y194, S196, Y197, N200, I201, L241, V242, E243, N245, L246, E243$^{(+)}$, T244$^{(+)}$, L246$^{(+)}$, L247$^{(+)}$, Y248$^{(+)}$, T249$^{(+)}$	P199, N200, I201, I202, L203, P204, M205, A237, A238, A239, L241, V242, H235, E243$^{(+)}$, M252$^{(+)}$, Y255$^{(+)}$, G256$^{(+)}$, A257$^{(+)}$, I259$^{(+)}$, F260$^{(+)}$, Y263$^{(+)}$	See Methods

C state	Intra1		Inter1	Inter2	Inter3	Pore
Residues:	P120, Q193, Y194, F195, Y197, I198, I202, N245, L246, Y254, Y255, I258	I201, I202, A239, V242, E243, Y254, Y255, G256, I258, I259	S196, Y197, I198, N200, I201, L241, V242, E243, N245, A239$^{(+)}$, I240$^{(+)}$, V242$^{(+)}$, E243$^{(+)}$, T244$^{(+)}$, L246$^{(+)}$,L247$^{(+)}$, Y248$^{(+)}$, T249$^{(+)}$, P250$^{(+)}$, Y251$^{(+)}$, G256$^{(+)}$, I259$^{(+)}$	Y197, P199, N200, I201, P204, A238, A239, L241, V242, E243, T244, N245, L246, I236$^{(+)}$, A239$^{(+)}$, I240$^{(+)}$, E243$^{(+)}$, L246$^{(+)}$, L247$^{(+)}$, Y248$^{(+)}$, G256$^{(+)}$, I259$^{(+)}$, F260$^{(+)}$	P120, Y194, S196, Y197, N200, I201, V242, N245, L246, L247$^{(+)}$, Y248$^{(+)}$, T249$^{(+)}$, P250$^{(+)}$	See Methods

Table S1 - List of residues used to constrain propofol within each site during FEP simulations. See Materials and Methods for details.

O state	Intra1	Intra2	Inter1	Inter2	Pore
Residues:	T255 (10±6%)<br>Y119 (8±6%)<br>I198 (7±4%)<br>T255 (5±3%)<br>Y197 (4±3%)	-	N200 (8±3%)<br>Y119 (6±4%)<br>N245 (6±2%)<br>V242 (6±3%)<br>I201(4±2%)	N200 (10±3%)<br>I201(9±3%)<br>T255 (6±4%)<br>L241 (4±2%)<br>I240 (3.3±0.7%)	L241 (3±2%)<br>A237 (0.9±0.8%)
H_2O:	17±7%	-	29±6%	15±6%	57±11%

C state	Intra1	Inter1	Inter2	Pore
Residues:	I201 (75±34%)<br>I202 (50±22%)<br>I206 (25±11%)<br>M205 (25±11%)	N200 (33±7%)<br>I201 (28±10%)	N200 (33±15%)<br>I201 (33±15%)	-
H_2O:	2±1%	38±9%	29±18%	17±18%

Table S2. Propofol -OH interactions from MTD simulations. Key residues involved in polar interactions are listed with the percent of time H-bonding interactions were seen with the propofol phenol -OH group. The cut-off distance for hydroxyl O atom to water H atom or hydroxyl H atom to water O atom was 1.725Å; backbone or side chain N-H group H atom to propofol hydroxyl O atom 1.77 Å; backbone or side chain carbonyl/carboxyl O atom to propofol hydroxyl H atom 1.875 Å; side chain hydroxyl O atom to propofol hydroxyl H atom 1.925 Å; and side chain N atom to propofol hydroxyl H atom 2.61 Å; based on analysis of radial distribution functions. Intra2 is not listed under the C state because it was not observed. No residues are listed for O state Intra2 or C state Pore because the propofol -OH group was not seen to form H bonds in these cases. Errors are standard error of means from 5 binding sites.

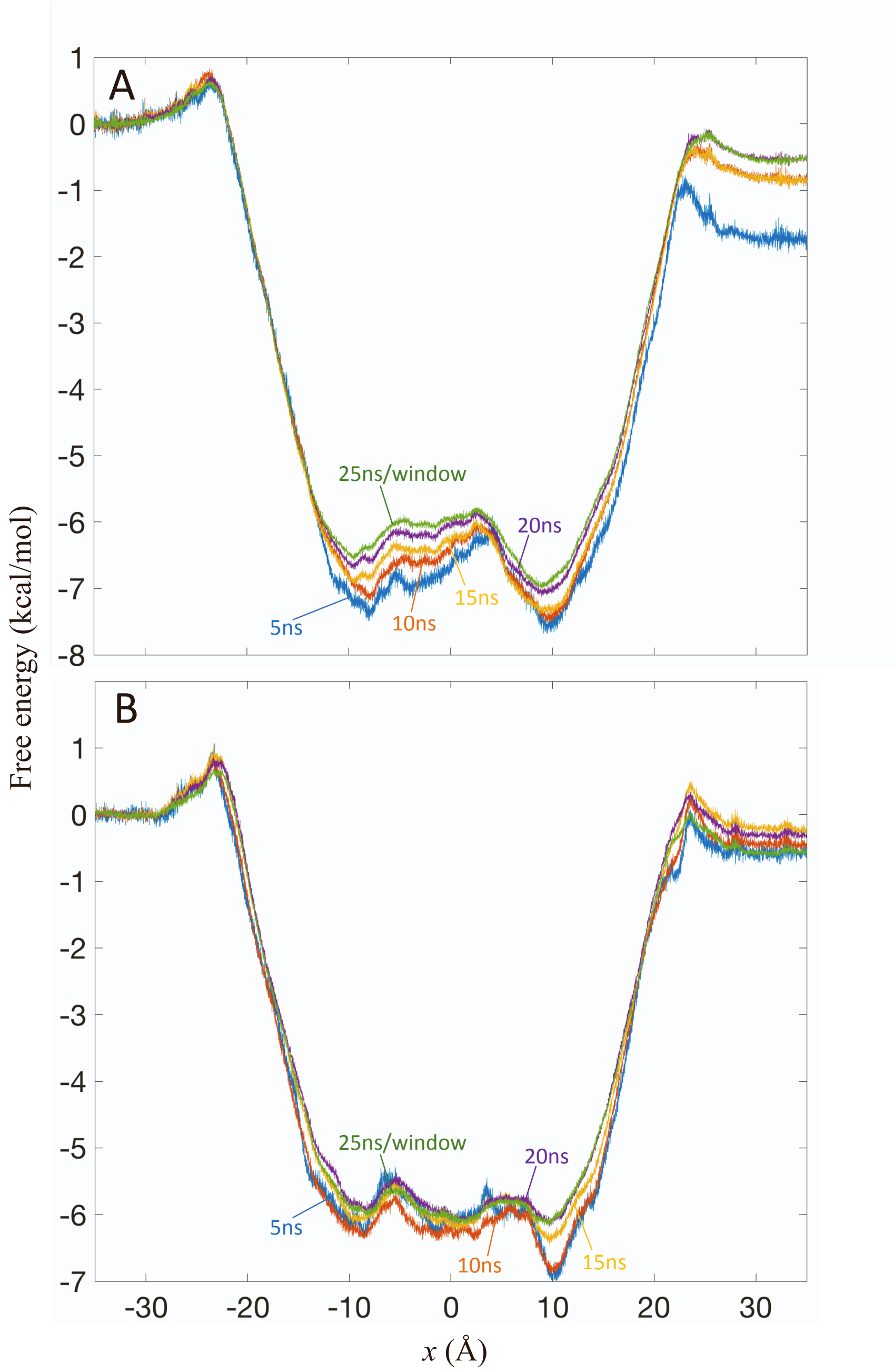

Figure S1. Free Energy Profiles for membrane translocation from Umbrella Sampling. A) propofol in a water/DPPC bilayer system; B) propofol in a water/POPC bilayer system; Each plot was formed from an incrementally longer simulation to demonstrate convergence in the results. Error bars (computed post-convergence) are not shown, but can be judged by the degree of asymmetry about the origin.

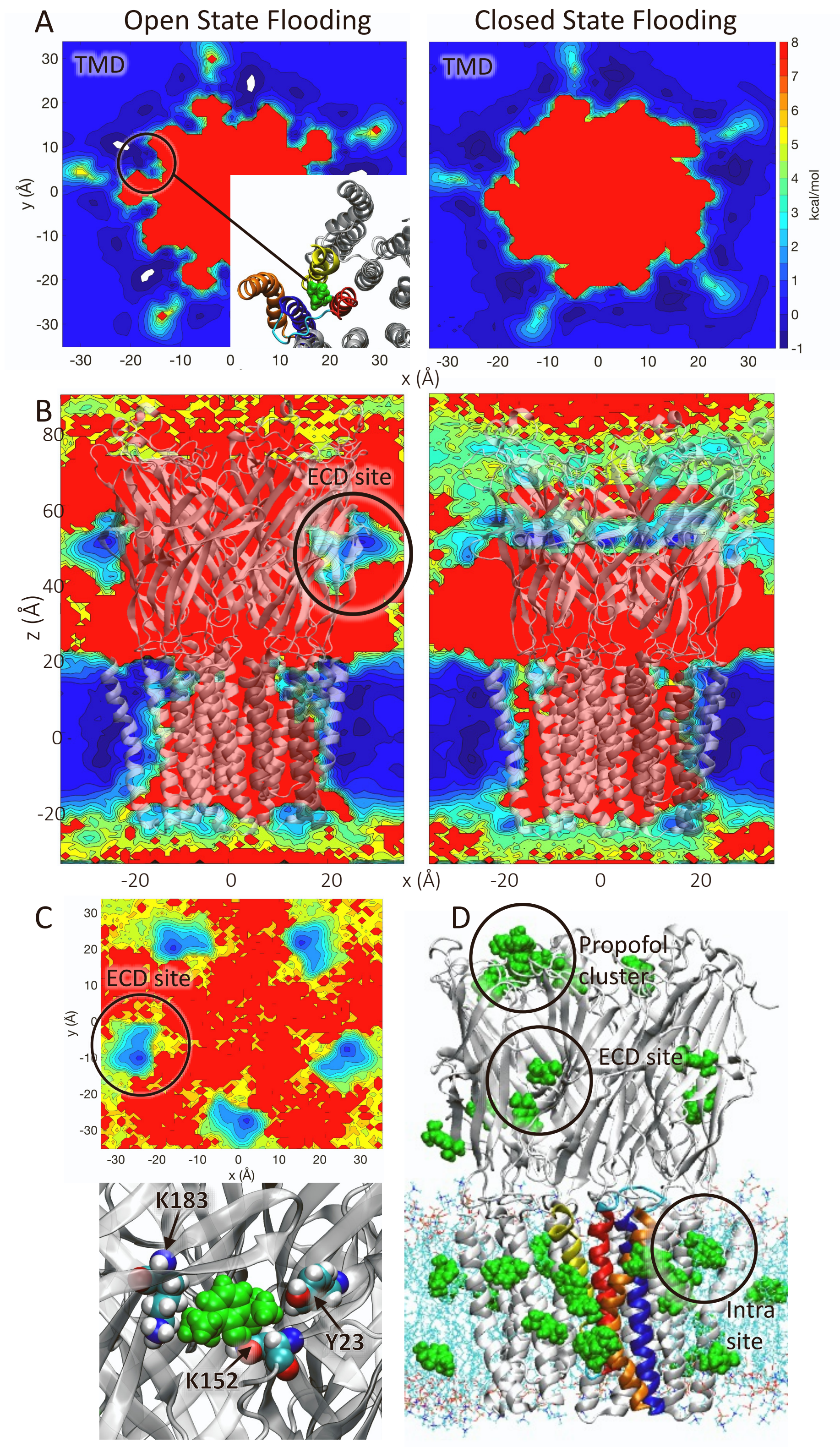

Figure S2: Unbiased exploration of propofol binding. A. *xy* projections of TMD PMFs generated from unbiased exploration. O state (left) C state (right). Intra-subunit site is visible in the O state. B) *xz* projections for the O state (left) and C state (right) with GLIC structure overlaid. The ECD binding minimum has been indicated. C) Propofol in the ECD site, with residues K183, N152 and Y23 visible. Y23 is on β1, while K183 is on β10 and K152 is on the β8-9 loop. D) Visualisation of a representative structure for O state GLIC, with a cluster of propofol adsorbed to the ECD, the ECD and Intra sites are occupied (from Fig.2).

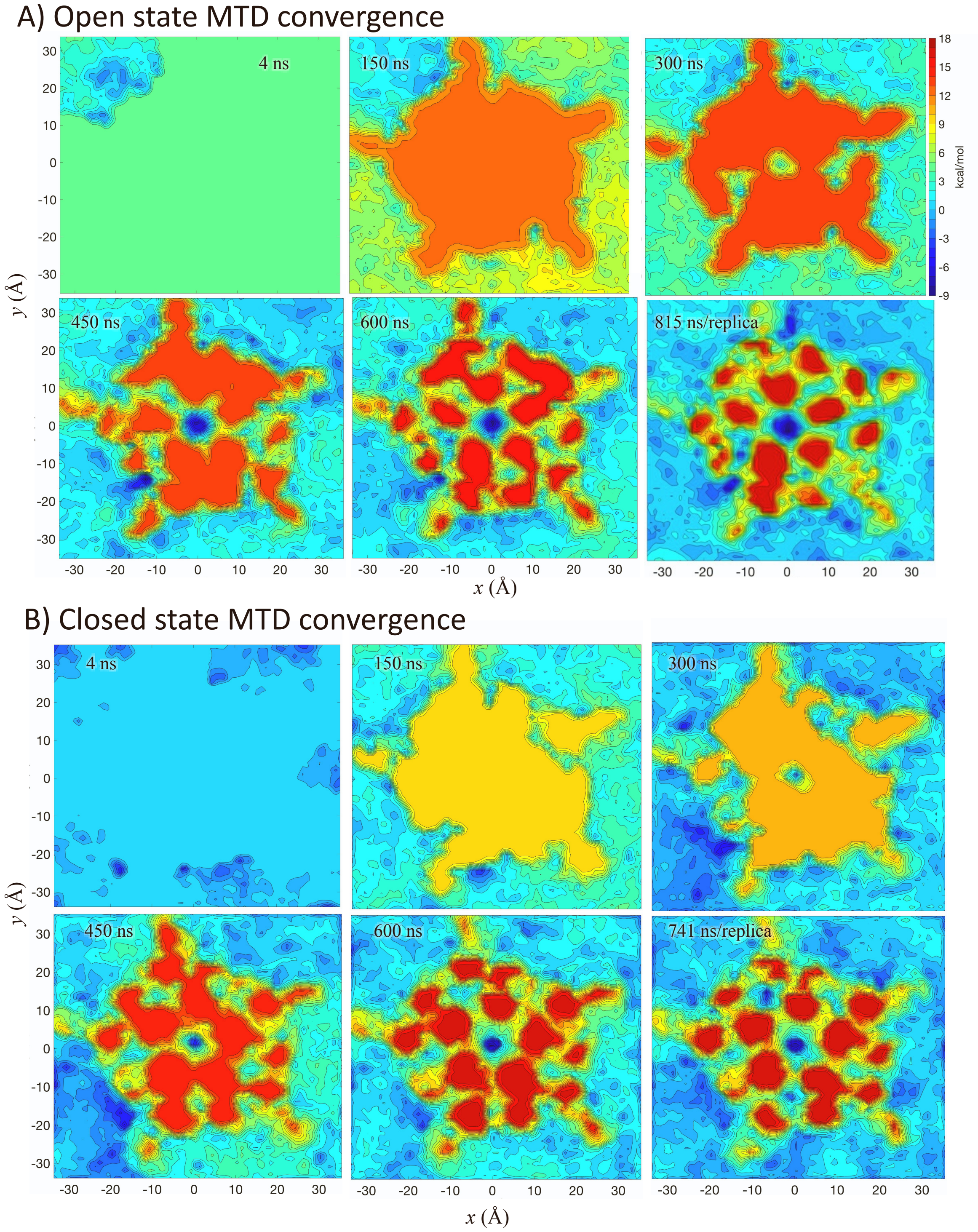

Figure S3. Convergence of MTD Free Energy Surfaces. Time series of O and C MTD exploration; A) *xy* projection of O state system as MTD progressed. You will note that the order of events observed are membrane exploration, visiting the intra site, travelling to the inter site and then entering the pore. This occurred in each subunit at different times. Eventually each subunit had distinguishable minima surrounded by a highly sampled membrane region. See also Video 1. B) *xy* projection of the C state as MTD progressed over time. You may notice that a uniform membrane estimation was achieved much earlier in the simulation due an even distribution of propofol molecules.

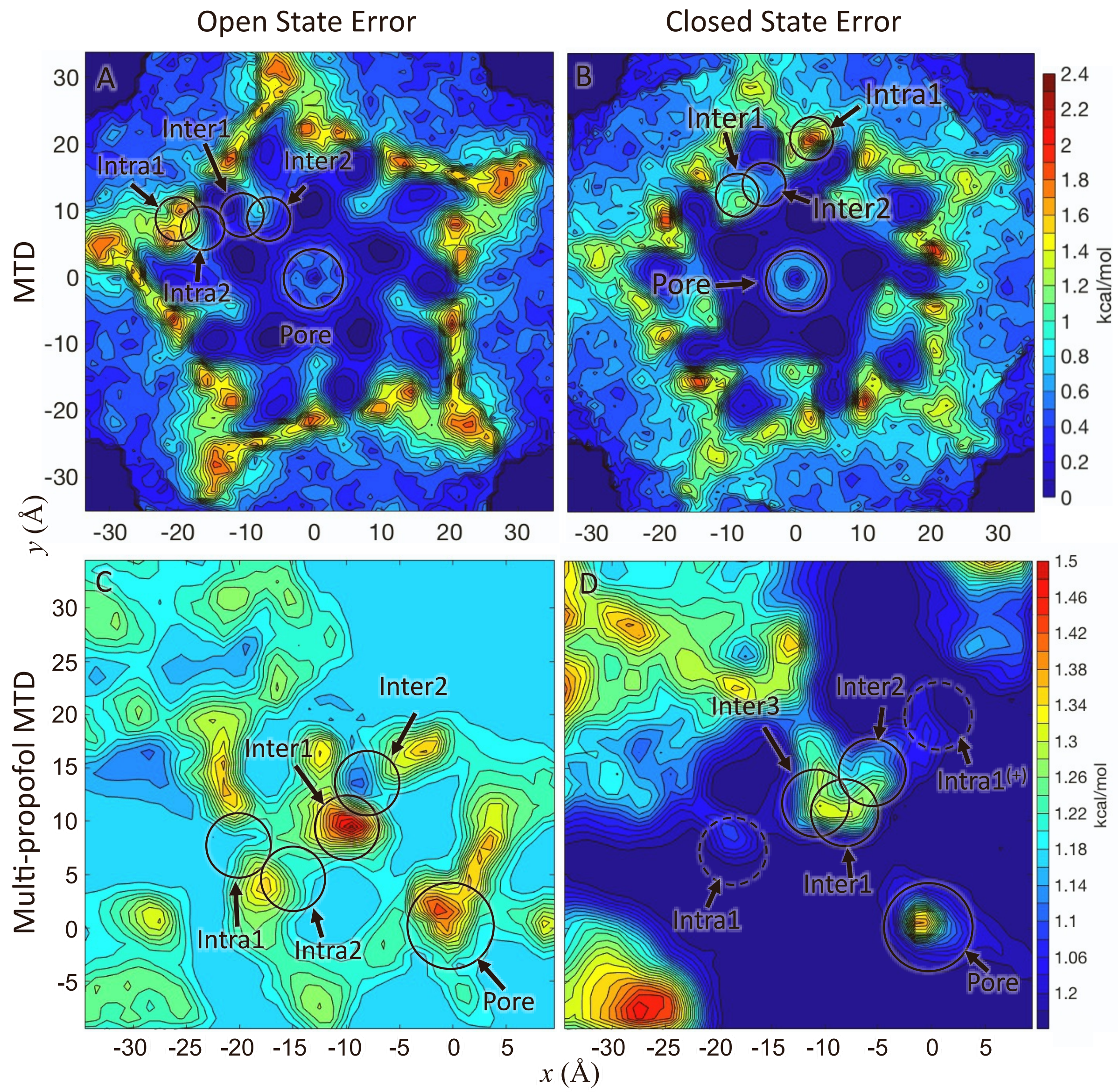

Figure S4. MTD free energy error analysis. The *xy* projection of error in the O (A) and C (B) states for the main MTD simulations based on asymmetry (see Materials and Methods). Error for the multi-propofol MTD is shown in C (O state) and D (C state). For this single-subunit free energy map, error was calculated using block averaging (see Materials and Methods).

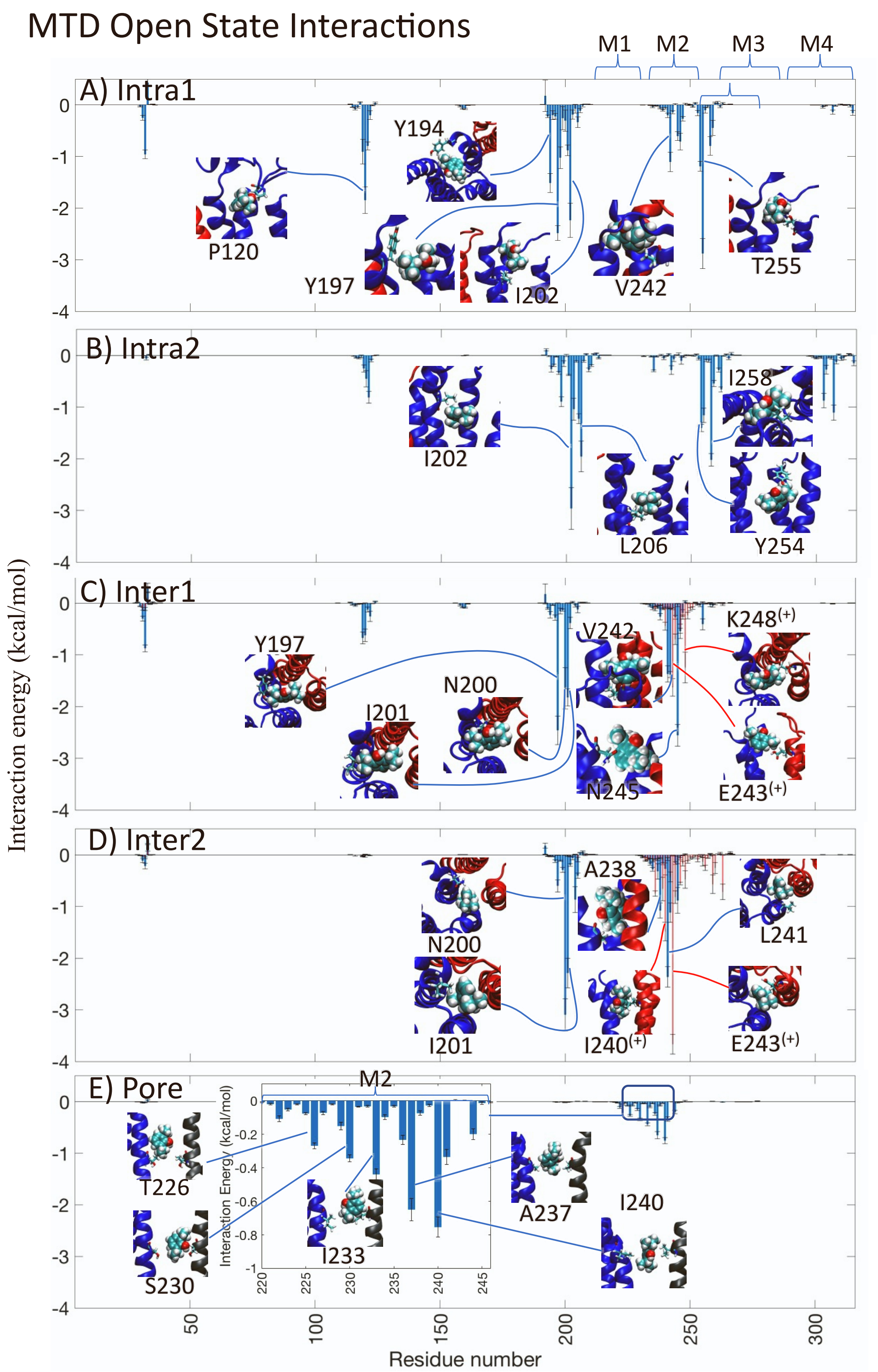

Figure S5. Propofol interaction energies within the O state from MTD. A) Inter1; B) Inter2; C) Intra1; D) Intra2; E) Pore. The (-) subunit of the site is coloured blue, while the (+) is coloured red. Intra sites occur inside a single subunit, so are only labelled in blue. Due to the lower mean interaction energies in the Pore, a zoomed-in inset has been shown.

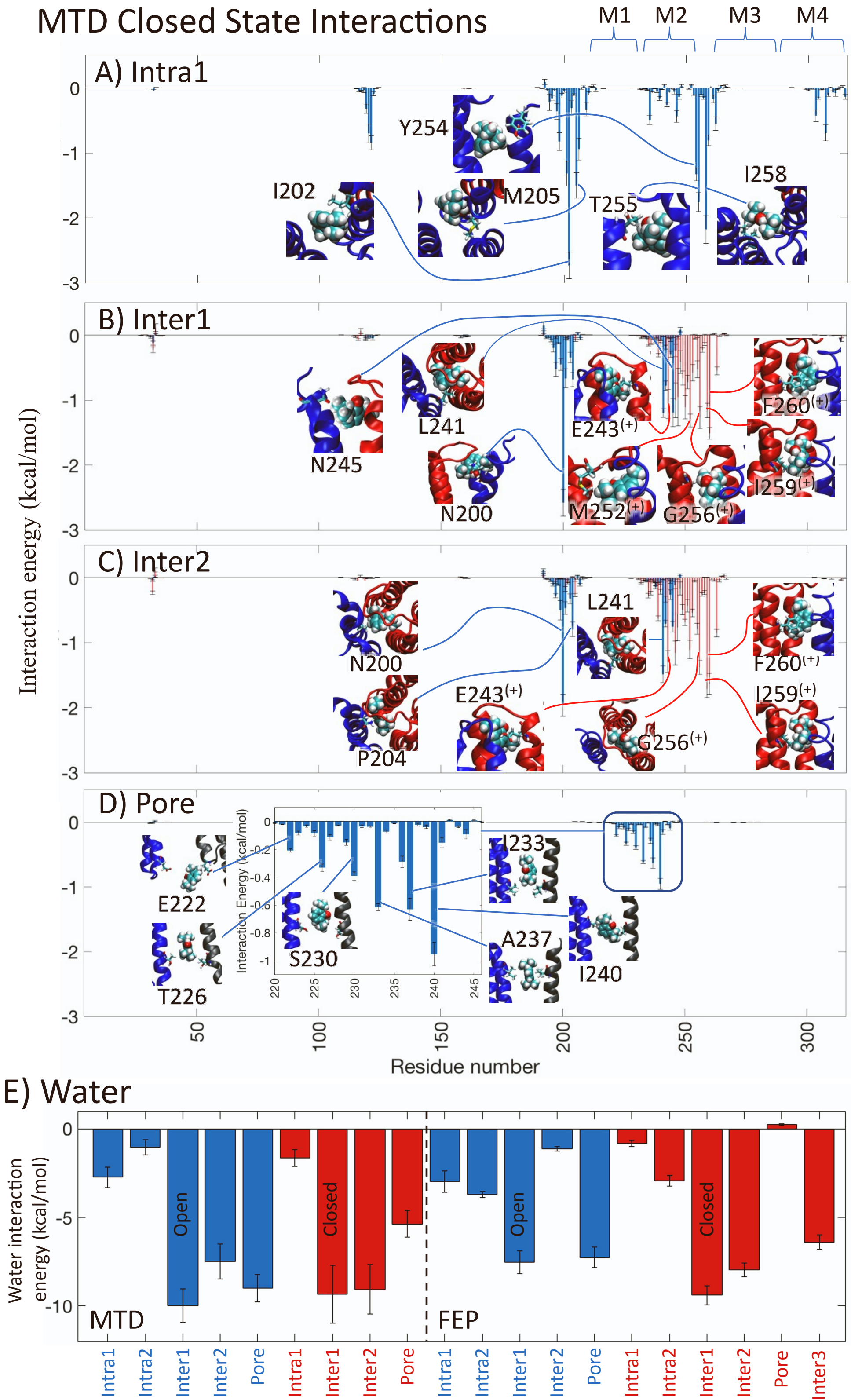

Figure S6. Propofol interaction energies within the C state from MTD. A) Inter1; B) Inter2; C) Intra; D) Pore. The (-) subunit of the site is coloured blue, while the (+) is coloured red. Intra sites occur inside a single subunit so are only labelled in blue. E) Interaction energies with water in all MTD (left) and FEP (right) simulations in both the O (blue) and C (red) states. *Pore indicates a restricted sampling of the Pore region in the C-state FEP simulations (see main text).

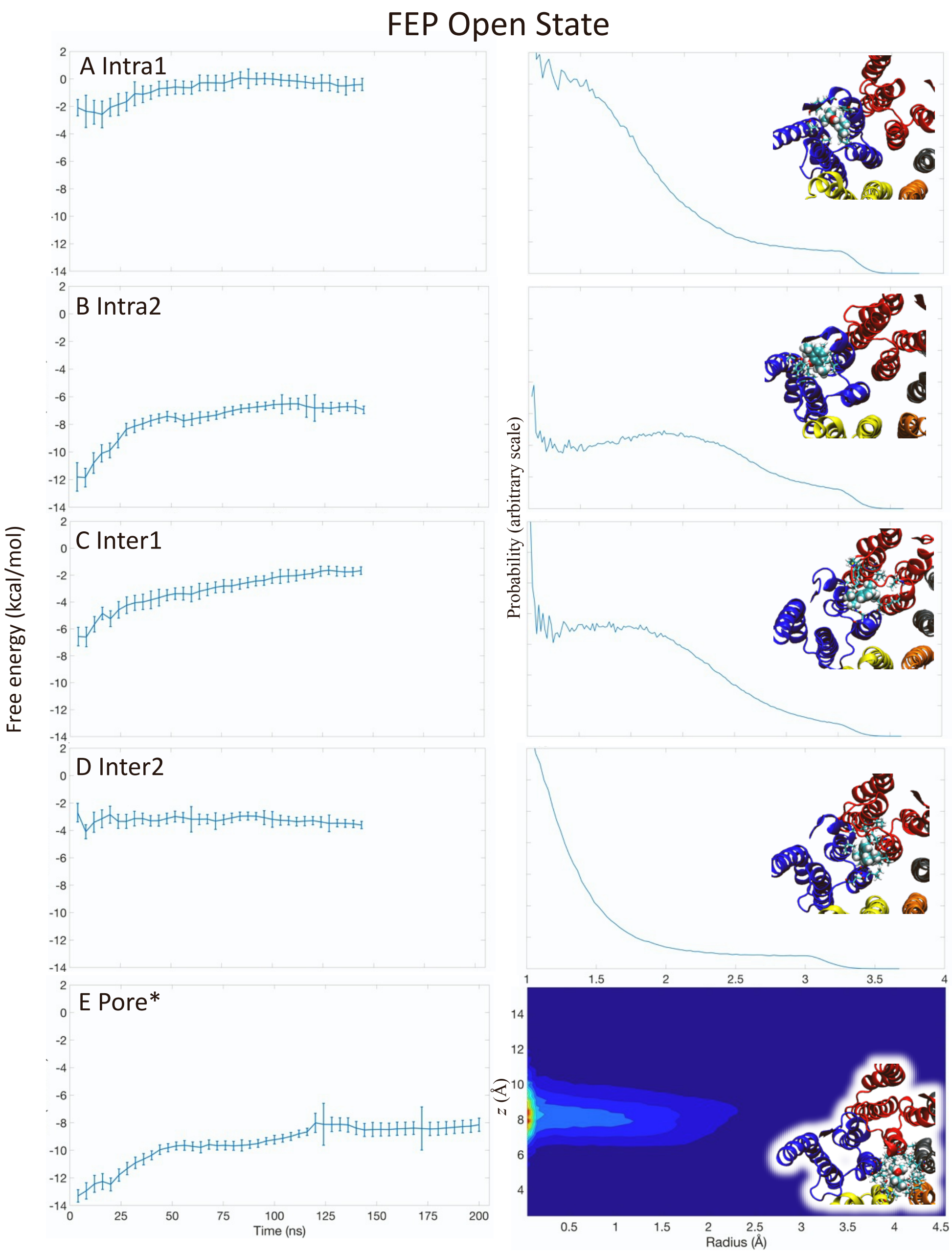

Figure S7. FEP convergence and site distributions for O state sites. Left - Free energy over time; Right – Radial distribution function within the site indicating the trapping of the propofol in a region centred on an energy minimum. These distributions allow one to determine if there exists a centred and clearly-defined binding site. Five sites investigated: A) Intra1; B) Intra2; C) Inter1; D) Inter2; E) Pore*. Pore histogram represented both radius and *z* position using a 2D heat map (asterisks indicating that only the upper pore is sampled in FEP).

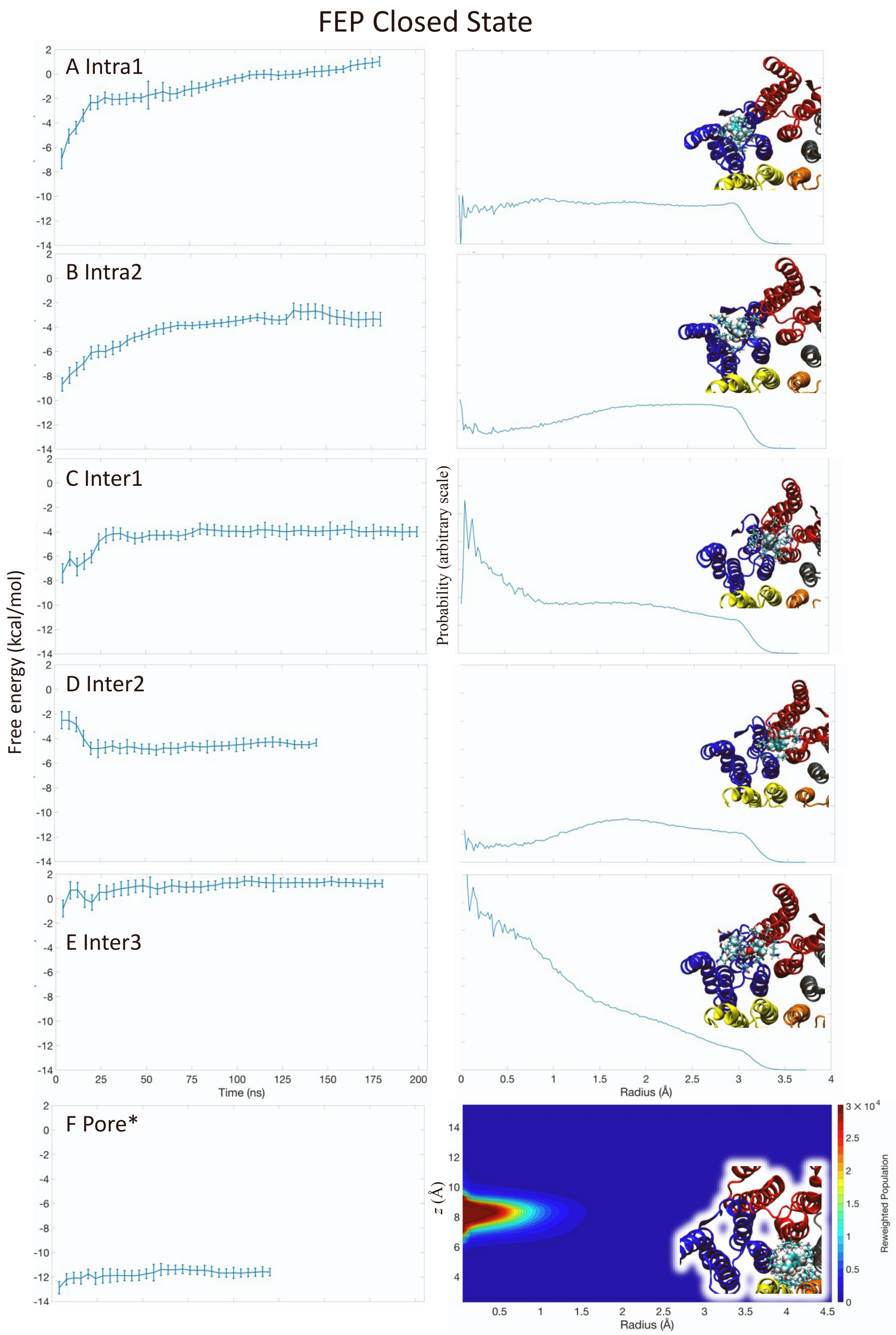

Figure S8. FEP convergence and site distributions for C state sites. Left - Free energy over time, revealing convergence of FEP for all except the Intra1 site. Right - Radial distribution function within the site, indicating the existence of a well-defined energy minimum within Inter sites, but less-well defined minima in the Intra sites (flatter distribution not centred on radius 0); noting that Intra1 was elevated in energy and Intra2 did not exist in the C state. The lack of a well-defined minimum for Intra1 explains the limited convergence in this region. A) Intra1; B) Intra2; C) Inter1; D) Inter2; E) Inter3; and F) Pore site. In F, the 2D map (right) shows that propofol was localised near the central axis in the upper pore site.

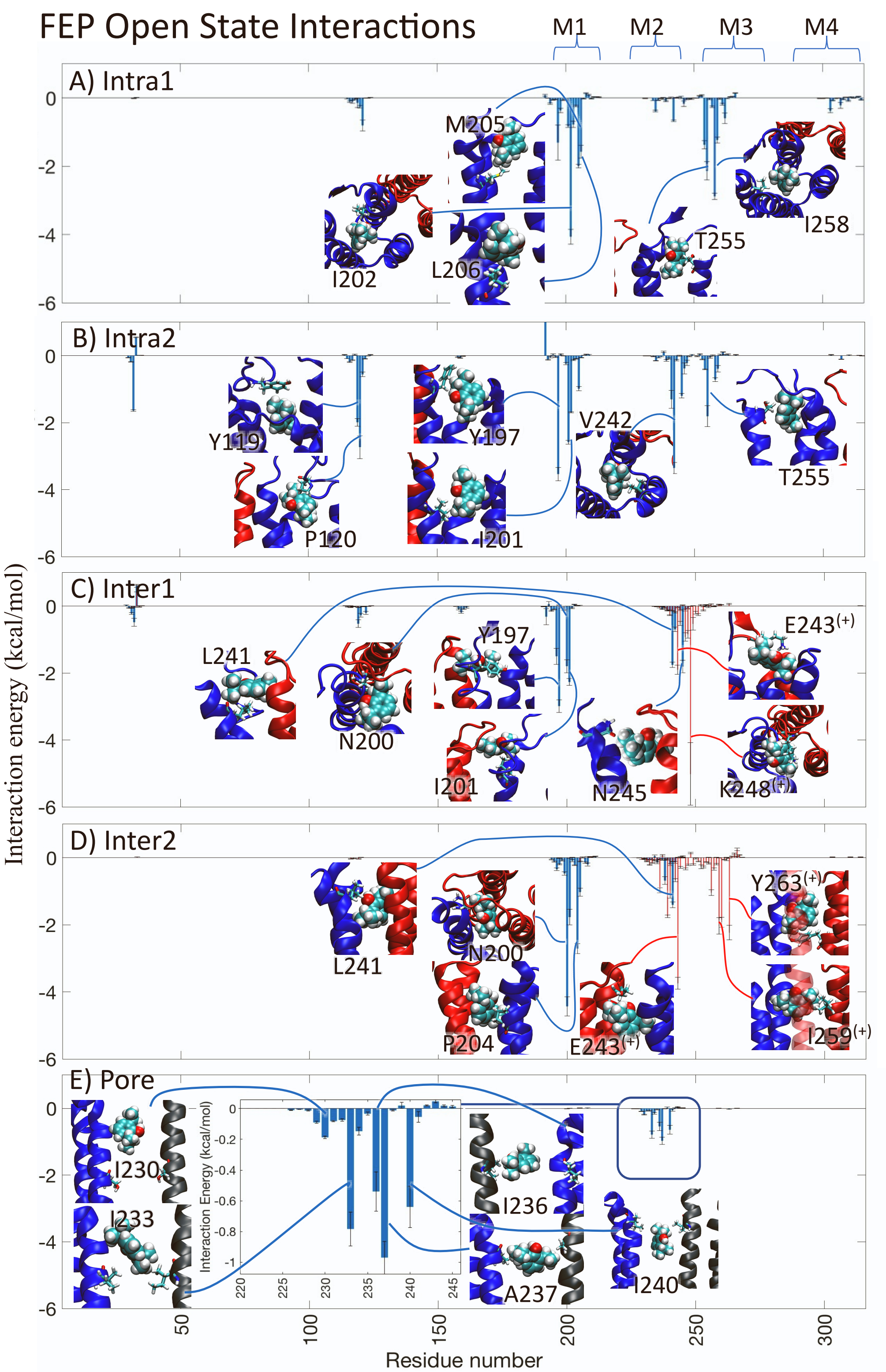

Figure S9. Propofol interaction energies within the O state from FEP. A) Intra1; B) Intra2; C) Inter1; D) Inter2; E) Pore. The (-) subunit of the site is coloured Blue, while the (+) is coloured red. Intra sites occur inside a single subunit so are only labelled in blue.

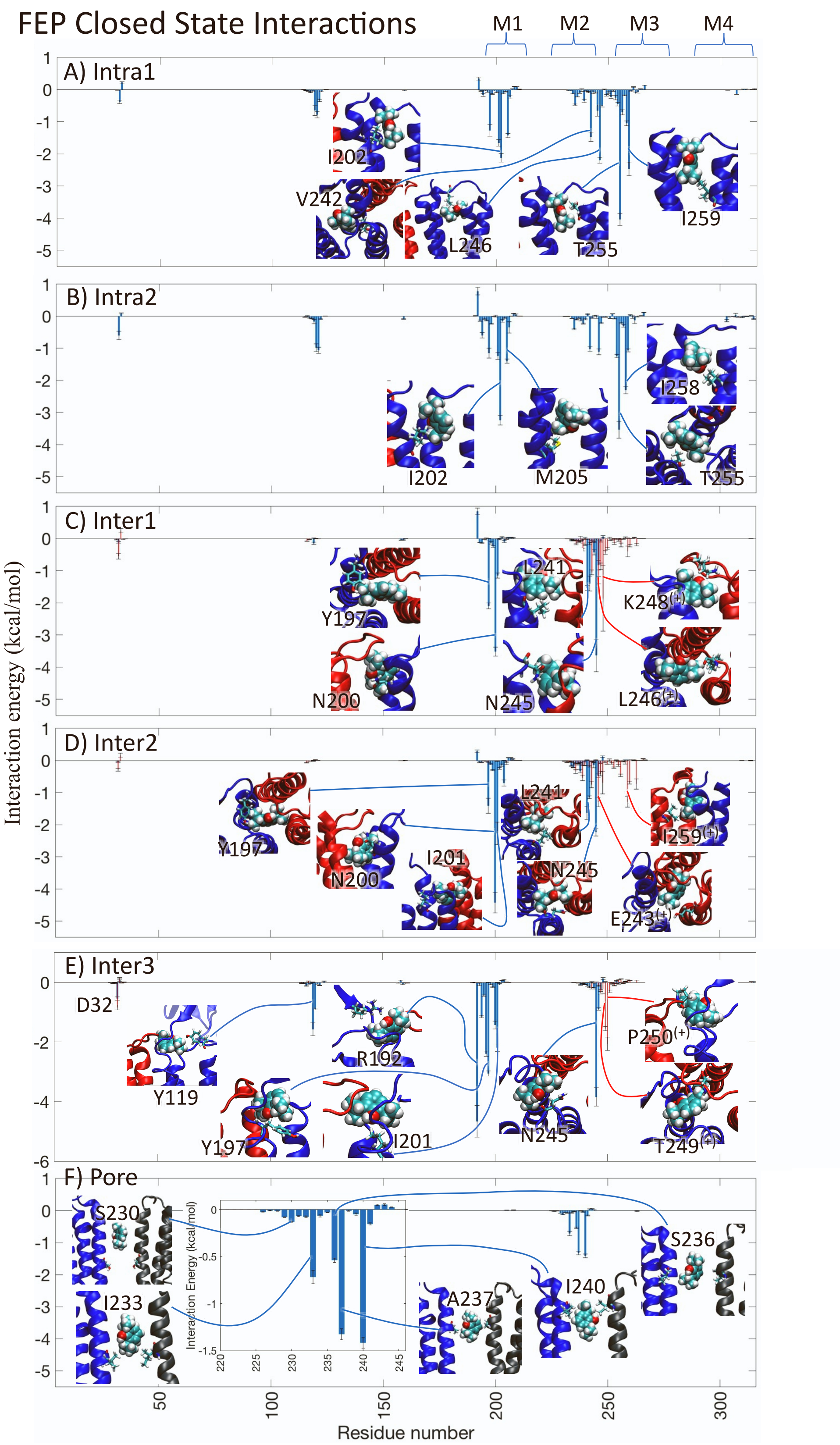

Figure S10. Propofol interaction energies within the C state from FEP. A) Intra1; B) Intra2; C) Inter1; D) Inter2; E) Inter3; and F) Pore. The (-) subunit of the site is coloured blue, while the (+) is coloured red. Intra sites occur inside a single subunit so are only labelled in blue.

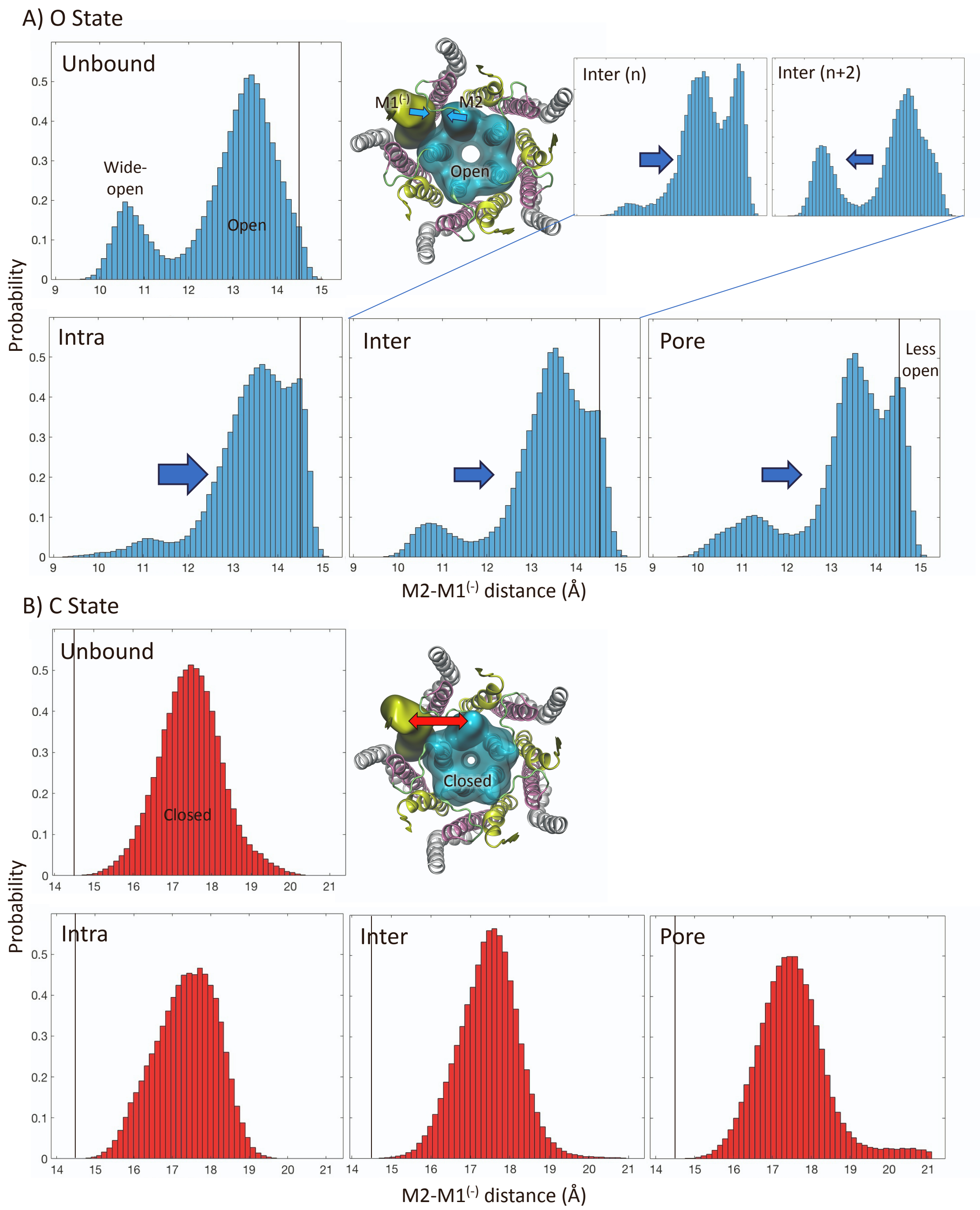

Figure S11. Effects of propofol binding on pore opening. A) Open state M2-M1$^{(-)}$ distances. The distribution without propofol unbound is shown, with values below ~11.5 considered 'wide open', and values between ~11.5 and ~14.5Å considered 'open'. Distributions with occupied Intra1, Inter1 and Pore sites are shown with arrows indicating change relative to the unbound case. Upper insets show distributions of M2-M1$^{(-)}$ distance within the same subunit and opposite (*n*+2) subunit space when the Inter site is occupied. B) Distributions without binding and with propofol bound to Intra1, Inter1 and Pore sites are shown, with all distributions having M2-M1$^{(-)}$ distances exceeding ~14.5Å and thus 'closed'. The flat-bottom restraint at 14.5 Å to maintain a conformation is indicated with a dashed vertical line.
